# Nuclear magnetic resonance therapy for deep-tissue biophysical stimulation: an integrated clinical and molecular analysis

**DOI:** 10.1093/bmb/ldag045

**Published:** 2026-11-09

**Authors:** Dalibor Krpan, Alireza Monajati, Aliya Yasmeen, Adam Benn, Ramy Atef Shenouda, Patrick R Hof, Markus Kipp, Tallal C Mamisch, Anna J Schreiner, Nicola Maffulli, Christoph Schmitz

**Affiliations:** Poliklinika K-CENTAR, Vrbik 8 a, 10000 Zagreb, Croatia; Department of Bioscience and Sport, School of Health Sport and Bioscience, University of East London, University Way, London E16 2RD, United Kingdom; MBST Physio and Joints Therapy Center, Dubai Healthcare City, Building 21 Block A, Umm Hurair 2, 00000 Dubai, UAE; Swave USA, LLC, 1100 Liberty Place, Sicklerville, NJ 08081, United States; Movement Clinic, D1 Building, Golf Central Mall, Palm Hills, Sheikh Zayed City, Cairo Governorate 3292531, Egypt; Nash Family Department of Neuroscience, Center for Discovery and Innovation, and Friedman Brain Institute, Icahn School of Medicine at Mount Sinai, 787 11th Avenue, New York, NY 10029, United States; Institute of Anatomy, Rostock University Medical Center, Gertrudenstr. 9, 18057 Rostock, Germany; Lifco AB, Verkmästaregatan 1, 74539 Enköping, Sweden; MedTec Medizintechnik GmbH, An der Hessenhalle 7, Gießen, Germany; Department of Orthopedics, Eberhard Karls University of Tuebingen, Hoppe-Seyler-Str. 3, 72076 Tuebingen, Germany; Department of Trauma and Orthopaedic Surgery, Faculty of Medicine and Psychology, University La Sapienza, Via di Grottarossa 1035, 00189 Roma, Italy; School of Pharmacy and Bioengineering, Keele University Faculty of Medicine, Thornburrow Drive, Stoke on Trent, ST4 7QB, United Kingdom; Centre for Sports and Exercise Medicine, Barts and the London School of Medicine and Dentistry, Mile End Hospital, Queen Mary University of London, 275 Bancroft Road, London E1 4DG, United Kingdom; Chair of Neuroanatomy, Institute of Anatomy, Faculty of Medicine, Ludwig-Maximilians-University of Munich (LMU Munich), Pettenkoferstr. 11, 80336 Munich, Germany

**Keywords:** nuclear magnetic resonance therapy, deep-tissue biophysical stimulation, mitochondrial bioenergetics and redox signaling, musculoskeletal regeneration, anti-inflammatory modulation

## Abstract

**Introduction or background:**

Musculoskeletal disorders such as osteoarthritis, chronic low back pain, radicular syndromes, and osteoporosis require noninvasive treatments reaching deep anatomical structures. Nuclear magnetic resonance therapy (NMRT/MBST) delivers depth-independent resonance-based stimulation. This systematic review consolidates clinical and mechanistic evidence to evaluate NMRT/MBST among deep-tissue biophysical therapeutics.

**Sources of data:**

A systematic search of PubMed, Ovid/Embase, and peer-reviewed literature identified 26 original studies spanning randomized and observational clinical evidence, imaging evaluations, veterinary trials, and *in vitro* research. Experimental findings on inflammatory, metabolic, mitochondrial, redox, and circadian pathways were integrated with clinical observations.

**Areas of agreement:**

NMRT/MBST shows a strong safety profile with no reported treatment-emergent adverse events. Across indications, studies demonstrate improvements in pain, function, and, selectively, structural or densitometric changes. Mechanistic evidence indicates modulation of mitochondrial bioenergetics, redox balance, inflammatory cascades, chondrogenic and osteogenic pathways, neurotrophic activity, and circadian regulation. These convergent processes mirror photobiomodulation, supporting NMRT/MBST as a deep-acting analog influencing regenerative biology in inaccessible tissues. Conventional pulsed electromagnetic field therapy is physically the closest comparator but shows narrower mechanistic overlap.

**Areas of controversy:**

Evidence remains heterogeneous in methodology, outcome measures, and structural responses. The only placebo-controlled knee osteoarthritis trial found no superiority over sham, although baseline imbalance limits interpretation.

**Growing points:**

The depth-independent profile and mechanistic parallels suggest applications in deep cartilage, subchondral bone, intervertebral discs, and nerve-root environments, supporting multimodal biophysical strategies.

**Areas timely for developing research:**

Priorities include larger randomized trials, longitudinal structural evaluation, comparisons with optical modalities, and biomarker-guided personalization.

## Introduction

Musculoskeletal and skeletal disorders, including osteoarthritis (OA), chronic low back pain, lumbar radicular syndromes, and osteoporosis, are leading causes of pain, disability, and healthcare utilization, especially in aging societies [1–4]. Conservative management commonly combines exercise therapy, education, analgesics/anti-inflammatory medication, injections, and, when needed, surgery [5–7]. However, long-term pharmacological strategies carry cumulative risk (e.g. gastrointestinal, renal, cardiovascular, metabolic) [8–10], and invasive interventions are not suitable or desired for many patients [3, 11, 12]. As a result, noninvasive physical medicine modalities have become increasingly relevant as adjuncts or alternatives within multimodal care pathways, particularly when they can reduce pain, improve function, and potentially influence underlying tissue biology [13–16].

Despite the breadth of these options, current standards of care remain limited in ways that are increasingly acknowledged at the guideline level. In OA, recommended pharmacological treatments provide only modest and often short-lived symptomatic relief; oral and topical nonsteroidal anti-inflammatory drugs (NSAIDs) are constrained by gastrointestinal, renal, and cardiovascular toxicity in the older, comorbid populations most commonly affected; intra-articular injections confer only transient benefit; and no agent has been shown to reliably modify structural disease progression. Joint arthroplasty, although effective, is invasive, has a finite implant lifespan, and is neither suitable nor desired for every patient [3, 6, 9]. In chronic low back pain and lumbar radicular syndromes, guidelines increasingly emphasize the limited and short-lived efficacy of analgesic pharmacotherapy, caution against long-term opioid use, and note that injections and surgery benefit only selected patients, leaving many with persistent symptoms despite guideline-concordant care [2, 5, 8, 11]. In osteoporosis, antiresorptive and anabolic agents reduce fracture risk but are limited by suboptimal long-term adherence, rare but serious adverse effects, and a persistent treatment gap, and they do not fully restore the compromised bone micro-architecture underlying fragility [4, 7, 10, 12]. A common thread across these conditions is that established care is largely symptom-oriented rather than tissue- and disease-modifying, is frequently constrained by cumulative safety concerns, and is often unable to reach or repair the deep structural compartments in which much of the underlying pathology resides.

A central practical limitation of many established noninvasive modalities is depth dependence. Techniques based on optical energy [e.g. low-level laser therapy/photobiomodulation therapy (PBMT), near-infrared laser therapy (NILT)] are constrained by absorption and scattering, which markedly reduce fluence with increasing depth of the target tissues and structures [17–19]. Mechanical modalities such as extracorporeal shock wave therapy (ESWT) can be highly effective for selected indications but are often focal, may be uncomfortable, and can be technically constrained for deep anatomical targets [13, 20, 21]. Conventional pulsed electromagnetic field therapy (PEMFT) can reach deeper than light, but frequently produces heterogeneous fields, and is not inherently designed around resonance coupling to ubiquitous biological nuclei [22–25]. These constraints matter because key pathological compartments in OA and spine disorders—including subchondral bone, deep cartilage zones, vertebral endplates, intervertebral discs, and nerve-root environments—are often located beyond the effective reach of surface-directed energy delivery [26–28].

Nuclear magnetic resonance therapy, also referred to as molecular biophysical stimulation therapy in the academic literature (NMRT/MBST), is positioned within this landscape as a noninvasive modality derived from nuclear magnetic resonance principles but operated at field strengths and radiofrequency (RF) energies far below those used for diagnostic magnetic resonance imaging (MRI) and far below thresholds associated with heating or ionizing effects [29, 30]. In NMRT/MBST, static magnetic fields are combined with low-frequency sweep fields and RF signals tuned to the resonance frequency of hydrogen nuclei [30]. Hydrogen protons—abundant in water and organic molecules—enter transient resonance states in which minuscule amounts of electromagnetic energy are absorbed and reemitted. This resonance-based interaction is commonly described in the NMRT/MBST context as adiabatic fast passage [31, 32], emphasizing the controlled traversal through resonance without thermal loading or mechanical impact.

A defining clinical feature of NMRT/MBST is that magnetic fields and the relevant low-frequency RF components are not meaningfully attenuated by biological tissues at therapeutic settings [33–35]. Consequently, NMRT/MBST can expose superficial and deep structures to comparatively uniform field penetration, enabling treatment of anatomical regions that are difficult to access with photonic or mechanical modalities. From a clinical “reach” perspective, this characteristic makes NMRT/MBST conceptually attractive for deep joints (e.g. hip), axial spine targets (e.g. discs, endplates, nerve roots), and skeletal compartments relevant to osteoporosis.

At the same time, contemporary rehabilitation medicine increasingly expects that noninvasive modalities should not only exert symptomatic benefits but also act through plausible biological mechanisms that align with tissue homeostasis: controlling inflammation, stabilizing cellular bioenergetics, and supporting anabolic repair signaling [3, 11, 14, 36, 37]. PBMT/NILT has an extensive mechanistic literature in this respect: photon absorption by mitochondrial chromophores (often highlighted: cytochrome c oxidase) can modulate respiration, transient redox signaling, inflammatory transcription factors, and growth-factor pathways [38–40]. Importantly, the responsiveness of cytochrome c oxidase to PBMT/NILT is not confined to a single wavelength. Early experimental work demonstrated robust activation at one investigated wavelength (670 nm) [38], whereas later research showed that comparable effects occur across multiple distinct red and near-infrared wavelengths (660 nm, 830 nm, and 905 nm) [39]. Together, these findings indicate that the key mechanistic determinant is adequate photon penetration rather than wavelength specificity per se—an important clarification for the present comparison. This raises an important translational question: can a resonance-based electromagnetic modality such as NMRT/MBST converge on similar intracellular endpoints—particularly those linked to mitochondrial function, redox balance, and inflammatory regulation—while bypassing the depth limitations of light?

Comparative analysis of NMRT/MBST and PBMT/NILT indicates substantial overlap in downstream biological effects across multiple domains relevant to musculoskeletal pathology: (i) preservation of cell viability with pro-proliferative tendencies in selected connective-tissue cell types such as chondrocytes and osteoblasts, alongside matrix-modulatory but nonproliferative responses in fibroblasts [41–48]; (ii) anti-inflammatory modulation including attenuation of nuclear factor kappa-light-chain-enhancer of activated B cells (NF-κB)–linked responses and suppression of cytokine-induced catabolic gene programs such as matrix metalloproteinases in chondrocytes [40, 42–44, 49, 50]; (iii) metabolic “recharging,” with improved cellular energy charge, reduced reliance on glycolysis, and stabilization of adenosine triphosphate (ATP) levels under stress, together with normalization of hypoxia-linked metabolic signaling [44, 45, 51–57]; and (iv) regenerative signaling extending to neuro-supportive actions including enhanced Schwann-cell–derived trophic support and neurite outgrowth in peripheral neural models [52, 53, 58–60]. Critically, this mechanistic comparison does not depend on enumerating specific PBMT/NILT wavelengths: while the initiating physics differ (photon–chromophore excitation versus magnetic-resonance/radical-pair mechanisms [54, 61, 62]), the canonical targets of PBMT/NILT—such as cytochrome c oxidase—are activated across a physiologically broad red/near-infrared spectrum [38, 39]. Therefore, wavelength specification is not mechanistically essential when comparing PBMT/NILT with NMRT/MBST at the level of downstream biology. Both modalities appear capable of shifting stressed tissues away from catabolic and inflammatory trajectories toward conditions supportive of repair and functional recovery [40–45, 51–55, 63].

This biological convergence is clinically relevant. Many of the most burdensome musculoskeletal disorders involve a coupled cycle of inflammation, metabolic stress, and impaired matrix maintenance. In OA, inflammatory cytokines drive catabolic enzyme expression, reduce anabolic transcriptional programs, and can impair chondrocyte energy homeostasis—mechanisms that align with pain, stiffness, and progressive structural deterioration [36]. In chronic spinal pain and radicular syndromes, peripheral nociception is shaped not only by mechanical compression but also by neuroinflammatory and metabolic factors within deep spinal compartments [64]. In osteoporosis, skeletal integrity is determined by bone remodeling balance within deep trabecular structures [4], where noninvasive stimulation is challenging. A modality that can reach deep tissues and plausibly modulate inflammation-metabolism-regeneration axes could therefore fill an unmet niche—provided clinical outcomes support meaningful patient benefit and safety.

This study addresses the same overarching topic—NMRT/MBST as a noninvasive therapy for musculoskeletal and skeletal disease—but from complementary vantage points. One synthesizes clinical outcomes across indications, emphasizing efficacy signals, durability, and safety in human and veterinary studies (summarized in Table 1). The other compares molecular and cellular mechanisms of NMRT/MBST (summarized in Table 2) with those of PBMT/NILT to clarify potential mechanistic overlap and to motivate the concept of NMRT/MBST as a “deep-penetrating” analog to PBMT/NILT-like stimulation in tissues inaccessible to light.

**Table 1:** Characteristics and main outcomes of human and veterinary studies evaluating NMRT/MBST.

Study	Design	Indication	Key findings	N _T_	N _C_	PEF	LF	Risk of bias assessment
[70]	Pre–post MRI structural study	Knee OA	Statistically significant increases in patellar cartilage thickness and volume and significant increases in multiple tibial cartilage parameters following NMRT/MBST.	14	0	10 weeks	10 weeks	High (noncontrolled pre–post design; small sample; objective imaging endpoints mitigate but do not remove bias)
[71]	Randomized, double-blind, placebo-controlled trial	Knee OA	Significant improvements occurred in both NMRT/MBST and placebo groups; NMRT/MBST did not demonstrate superiority over placebo at 12 weeks across pain/function measures and imaging endpoints (ultrasound cartilage thickness; MRI WORMS).	50	50	12 weeks	12 weeks	Low (strong RCT methodology with blinding and imaging endpoints; neutral result reduces risk of overestimation)
[72]	Long-term questionnaire-based follow-up survey	Knee OA	Survey-based evidence of sustained improvement in pain and daily-life functional limitations up to 4 years after NMRT/MBST, with slight pain increase toward the end of the observation period.	39	0	4 months	Up to 4 years	High (survey-based self-report and recall bias; no comparator; long follow-up provides valuable durability signal)
[73]	Large multicenter longitudinal observational survey	Degenerative rheumatic disease (OA and spinal pain)	Sustained improvements in pain and function across multiple indications, with benefits persisting through 12 months.	≈4500	0	3–12 months	12 months	High (observational design without comparator; large sample and consistent longitudinal trends strengthen plausibility, but confounding remains)
[77]	Randomized, blinded, sham-controlled *in vivo* animal study (ACL-transection rabbit model)	Post-traumatic knee OA induced by bilateral ACL transection in rabbits	Early NMRT/MBST initiation (6 weeks post-transection) significantly reduced macroscopic OA severity compared with sham (*P* < .01), driven by fewer fibrillations, reduced osteophyte formation, and less joint effusion. No macroscopic improvement when treatment was initiated at 12 weeks. No histological improvement (Mankin score) at either timepoint.	8 + 8	4 + 4	One week after completion of treatment^a^	14 weeks after ACL transection (end of evaluation period)	Moderate risk of bias. Strengths include randomization, blinded macroscopic assessment, and sham-treated controls. Limitations include small group sizes, differing exclusions between groups, absence of blinding for histology explicitly stated, and potential confounding due to ongoing joint instability after ACL transection.
[78]	Randomized, placebo-controlled clinical trial	Finger-joint OA	Significant and sustained improvements in pain and hand function in the NMRT/MBST group; placebo group showed no improvement and deterioration in some measures at 6 months.	35	35	6 weeks	6 months	Low-to-moderate (placebo-controlled RCT; modest sample size; outcome measures include validated pain/function scales)
[79]	Randomized, double-blind, placebo-controlled inpatient rehabilitation trial	Chronic low back pain	Greater durability of pain reduction in the NMRT/MBST group versus placebo at 3 months; improvements also observed in disability-related measures.	31	31	3 months	3 months	Moderate (controlled design supports internal validity; inpatient context and limited follow-up may influence generalizability)
[80]	Randomized, double-blind, placebo-controlled inpatient rehabilitation trial	Chronic low back pain	Active NMRT/MBST and placebo groups both improved under standardized inpatient rehabilitation; NMRT/MBST showed more consistent advantages in dynamic pain (pain under stress) and selected disability domains (e.g. personal care), with higher proportions reporting improvement and fewer deteriorations. Sleep and walking improved similarly across groups at follow-up.	31	31	1 week and 3 months	3 months	Moderate (controlled, blinded inpatient trial with clinically relevant outcomes; several domains show NMRT/MBST advantages, but improvements in both groups and inpatient context affect generalizability)
[81]	Randomized, double-blind, placebo-controlled trial	Nerve-root irritation following lumbar disc pathology	Significant reduction in sick-leave days in the NMRT/MBST group versus placebo, with group differences more apparent at later follow-up points.	54	54	12 weeks	12 weeks	Low-to-moderate (double-blind RCT; modest sample; pragmatic endpoint reduces subjectivity, but details may vary by subgroup)
[82]	Randomized, double-blind, placebo-controlled trial	Lumbar disc herniation with lumbar radicular syndrome	Both groups improved over time; NMRT/MBST showed a statistically significant advantage in pain at week 4 and significantly fewer sick-leave days at 3 months; MRI morphology improved similarly in both groups.	54	54	12 weeks	12 weeks	Low (double-blind RCT with sham control and predefined outcomes; some endpoints show limited between-group separation)
[83]	Prospective pre–post densitometric study	Osteoporosis	Statistically significant improvements in bone mineral density (T-scores) at lumbar spine and hip regions at 12 months.	103	0	12 months	12 months	Moderate (objective DXA outcomes; absence of control group limits causal attribution)
[84]	Retrospective longitudinal cohort with case descriptions	Osteoporosis	Long-term follow-up describing severe trauma events without fracture in selected cases after NMRT/MBST; cohort followed up to 5 years.	450	0	1–3 months	Up to 5 years	High (retrospective uncontrolled design; potential selection/reporting bias; clinically meaningful endpoint but not systematically compared)
[85]	Long-term veterinary case report	Canine hip OA secondary to dysplasia	Delayed but substantial functional improvement after NMRT/MBST, with reproducible benefit after retreatment and sustained mobility with yearly cycles over 9 years.	1	0	~3 months	9 years	Very high (single uncontrolled case; narrative outcome assessment; nevertheless, minimizes human placebo explanations)
[86]	Controlled veterinary trial with objective locomotor outcomes	Canine elbow OA	Objective gait, lameness, pain, and range-of-motion measures were largely stable in both groups. The MBST/NMRT group showed numerically favorable trends—such as lower symmetry indices and improved range of motion at 3 months—while the placebo group tended to remain unchanged or decline. Although differences were not statistically significant, overall treatment-effectiveness patterns indicate that some MBST/NMRT-treated dogs showed clinically relevant improvements, suggesting a modest functional benefit in canine elbow OA.	14	14	3 months	6 months	Moderate (controlled veterinary setting; objective endpoints strengthen reliability; sample size and heterogeneity may limit precision)
[87]	Randomized, double-blinded, placebo-controlled veterinary trial	Canine OA (various joints)	NMRT/MBST produced significant within-group improvements in vertical impulse symmetry (SI_IFz) and lameness at 3 months, while placebo showed no comparable change. Between-group differences were not statistically significant, but overall treatment effectiveness scores favored MBST at all follow-up points. Improvements diminished by 6 months; pain scores did not differ between groups.	15	15	3 months	6 months	Moderate (rigorous blinding and objective gait metrics strengthen validity; small sample, dropouts, heterogeneous OA sites, and allowance of concomitant treatments limit interpretability).

^a^Assessed 1 week after completion of the 7-day treatment series (i.e. 8 weeks post-ACL transection for early-treatment and matched control groups, and 14 weeks post-ACL transection for late-treatment and matched control groups).

Abbreviations: ACL, anterior cruciate ligament; DXA, dual-energy X-ray absorptiometry; NT, number of patients in the treatment group; NC, number of patients in the control group; PEF, primary endpoint follow-up; LF, longest follow-up; MRI, magnetic resonance imaging; OA, osteoarthritis; RCT, randomized controlled trial; WORMS, whole-organ magnetic resonance imaging score.

**Table 2:** Summary of experimentally demonstrated molecular mechanisms of NMRT/MBST across cell systems.

Mechanistic domain	Specific effects observed	Model systems/cell types	Supporting studies
Anti-inflammatory effects	Suppression of IL-1β–induced NF-κB activation Downregulation of catabolic cytokines (IL-6, IL-8) Reduction of matrix-degrading enzymes (MMP-13, MMP-3)	Osteoarthritic chondrocytes	[42–44]
Cell proliferation and viability	Strong increase in osteoblast and chondrocyte proliferation Preservation of viability; no apoptosis induction	Osteoblasts, chondrocytes	[41–43]
Anabolic/ECM maintenance pathways	Restoration of IGF-, EGF-, and FGF-related signaling Preservation of integrins (ITGA3, ITGB1) Maintenance/upregulation of cartilage-specific collagens (e.g. COL9A1)	Chondrocytes	[42, 43]
ECM remodeling (nonproliferative)	No effect on cell proliferation or viability Redistribution of collagen solubility fractions Altered ECM organization without cytotoxicity	Human dermal fibroblasts	[45]
Neuronal growth and support	Increased neurite length and branching in DRG neurons Enhanced Schwann-cell–mediated neuro-supportive signaling Improved neuronal mitochondrial metabolism	DRG neurons, Schwann cells	[52, 53]
Mitochondrial function/energy homeostasis	Increased ATP availability Reduced reliance on glycolysis Stabilization of mitochondrial respiration Improved NAD^+^/NADH balance	Chondrocytes, Schwann cells, fibroblast clock reporters	[43, 44, 52–54]
Hypoxia-related signaling	Normalization of HIF pathway responses (HIF-1α, HIF-3α) Prevention of hypoxia-induced metabolic dysfunction Restoration of hypoxia-related circadian responses	Zebrafish fibroblasts and larvae, chondrocytes, NIH3T3 clock cells	[44, 51, 52, 54, 61]
Redox regulation	Controlled redistribution of mitochondrial H₂O₂ and O₂•^−^ Prevention of pathological NADH accumulation Maintenance of redox oscillations	NIH3T3 fibroblasts (Per2:Luc), chondrocytes	[44, 54]
Radical-pair/magnetosensitive mechanisms	Effects abolished when superoxide is quenched Catalase prevents excessive ROS while preserving signaling Behavior consistent with radical-pair activity in cryptochrome	NIH3T3 cells, circadian clock systems	[54, 61, 62]
Epigenetic regulation	Modulation of microRNAs regulating inflammation and ECM repair (miR-27, miR-140, miR-146a etc.) Alteration of HDAC4 activity Reprogramming of transcriptional networks	Osteoarthritic chondrocytes	[44]
Circadian clock regulation	Modulation of Per2, Cry1/2, Bmal1 oscillations Day/night-dependent effects under hypoxia Integration of metabolic and redox oscillations	NIH3T3 Per2:Luc cells, zebrafish cells and larvae	[51, 54, 61, 62]

Abbreviations: ECM, extracellular matrix; DRG, dorsal root ganglion; ROS, reactive oxygen species.

Accordingly, the objective of this integrated study is to unify the clinical evidence on NMRT/MBST across musculoskeletal and skeletal indications, and the mechanistic rationale emerging from comparative cellular biology, into a single coherent narrative. Specifically, we aim to (i) summarize the breadth and structure of the clinical evidence base (trial designs, indications, outcomes, follow-up horizons and safety); (ii) align clinical endpoints (pain, function, imaging, densitometry, work-related outcomes) with plausible biological mechanisms (inflammation control, metabolic stabilization, tissue-regenerative signaling); and (iii) identify research gaps and the next experimental/clinical steps needed to validate the role of NMRT/MBST—alone or in combination with PBMT/NILT and other rehabilitation modalities—within conservative management pathways for deep and superficial musculoskeletal (as well as central nervous system) ailments.

## Materials and methods

A systematic search was performed in PubMed and Ovid/Embase (including all resources) using the terms “MBST,” “MBST therapy,” “molecular biophysical stimulation therapy”, “NMRT,” “nuclear magnetic resonance therapy,” “tNMR,” and “therapeutic nuclear magnetic resonance” from database inception through 28 December 2025, in accordance with the 2020 PRISMA (Preferred Reporting Items for Systematic Reviews and Meta-Analyses) guidelines [65]. The assessment strategy for the identified records is summarized in Fig. 1.

**Figure 1:**
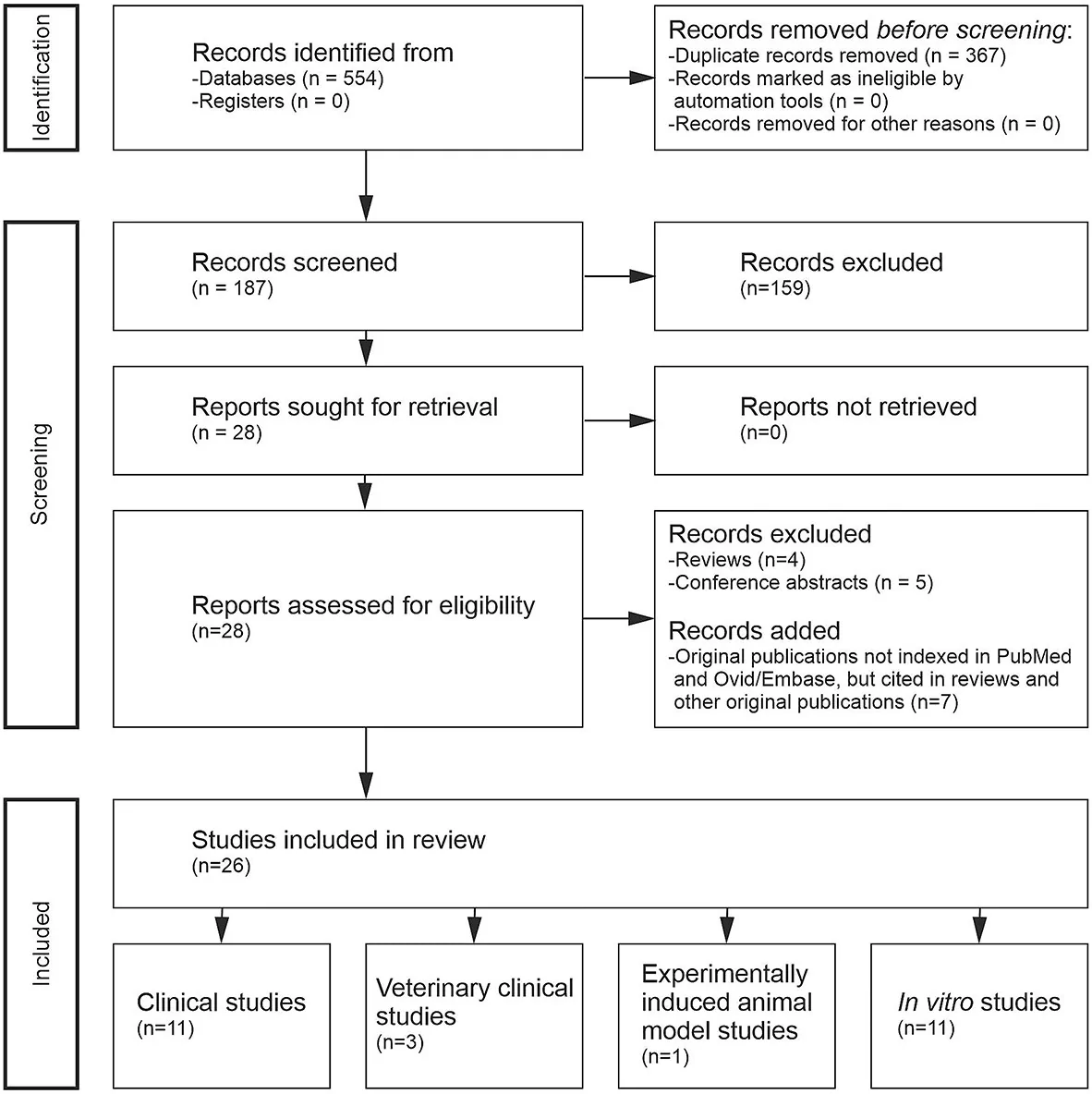
Systematic review flow chart of the literature search regarding NMRT/MBST, performed according to the PRISMA guidelines [65] on 28 December 2025.

In PubMed, the searches retrieved 62 records for “MBST,” 28 for “MBST therapy,” 2 for “molecular biophysical stimulation therapy,” 26 for “NMRT,” 10 for “nuclear magnetic resonance therapy,” 16 for “tNMR,” and 8 for “therapeutic nuclear magnetic resonance.” In Ovid/Embase, the same searches retrieved 178, 62, 4, 72, 35, 35, and 16 records, respectively. Across both databases, this yielded a total of 554 records, from which 367 duplicates were removed prior to screening. No automated tools were used to flag records as ineligible.

The remaining 187 records were screened, and 159 were excluded because they were unrelated to NMRT/MBST; this substantial number reflects the common use of the abbreviations “MBST,” “NMRT,” and “tNMR” in unrelated scientific fields (details in Tables A1 and A2 in the Appendix). All 28 records sought for retrieval were obtained either from the E-media library of LMU Munich (Munich, Germany) or other sources and were subsequently assessed for eligibility. Publications in languages other than English were translated using ChatGPT (version 5.2; OpenAI OpCo, San Francisco, CA, USA).

Among the 28 retrieved records, five were conference abstracts and were excluded. The remaining publications consisted of 19 original articles and four peer-reviewed reviews [66–69]. Screening the reference lists of these 19 original articles and four reviews identified seven additional peer-reviewed original articles. The final dataset therefore comprised 26 peer-reviewed original articles, including 11 clinical studies, three veterinary clinical studies, one experimentally induced animal model study, and 11 *in vitro* studies. None of these studies were excluded from the systematic review based on study outcome.

Given the heterogeneity of clinical indications addressed in the included clinical studies, no statistical meta-analysis was performed.

## Results

### Clinical outcomes across indications

#### Knee osteoarthritis

Clinical investigation of NMRT/MBST in knee OA includes early structural MRI studies [70], one randomized controlled trial [71], and long-term observational follow-up [72, 73]. Initial pre–post MRI work demonstrated statistically significant increases in cartilage thickness and volume following NMRT/MBST, with changes observed in both patellar and tibial compartments [70]. Resonance-based stimulation could therefore influence cartilage morphology, although interpretation is limited by the absence of a control group in the latter study [70].

A subsequent randomized, double-blind, placebo-controlled trial provided a more conservative perspective [71]. Over a 12-week treatment period, both active and sham groups demonstrated improvements in pain, functional scores and imaging parameters assessed by ultrasound and MRI-based scoring systems. No significant differences between groups were detected at the primary endpoint. This trial, however, warrants strong reservation. The two groups differed significantly at baseline in the primary outcome: the NMRT/MBST group reported significantly higher pain than the sham group (VAS 6.4 versus 5.5; *P* = .045), an imbalance that was not accounted for in the analysis. Both groups reached almost identical pain scores at 12 weeks (VAS 3.8 versus 3.6), but the more severely affected NMRT/MBST group experienced the larger absolute improvement (a mean reduction of 2.6 versus 1.9 points), so that a baseline-adjusted analysis, such as analysis of covariance, could have yielded a different conclusion from the unadjusted between-group comparison reported. Importantly, treatment was well tolerated, and no safety concerns emerged [71]. Short-term symptomatic improvement can occur therefore in knee OA without clear between-group separation, a pattern consistent with the substantial placebo responsiveness known in this condition [74–76].

Preclinical evidence in knee OA also supports early-phase NMRT/MBST-mediated modulation of degenerative processes. In a blinded, randomized, rabbit anterior cruciate ligament (ACL)-transection model of post-traumatic OA, NMRT/MBST initiated 6 weeks after ACL transection resulted in significantly lower macroscopic OA severity compared with sham-treated controls [77]. Improvements were primarily driven by reduced osteophyte formation, fewer fibrillations of the articular cartilage, and decreased joint effusion. When NMRT/MBST was initiated later, at 12 weeks post-injury, no macroscopic differences were observed, and in both timing groups, histologic Mankin scores were not altered. Hence, NMRT/MBST may preferentially influence early inflammatory and structural responses preceding more advanced cartilage matrix deterioration [77].

#### Finger-joint osteoarthritis

Evidence for small-joint OA demonstrates a clearer treatment signal. In a randomized, placebo-controlled trial, patients with finger-joint OA who received NMRT/MBST experienced significant and sustained reductions in pain alongside marked improvements in hand function [78]. These benefits persisted through mid-term follow-up. In contrast, placebo-treated patients showed minimal improvement and, in some functional domains, progressive deterioration over time [78]. The magnitude and durability of benefit observed in this setting indicate a robust therapeutic effect of NMRT/MBST in small-joint degenerative disease.

#### Chronic low back pain

Chronic low back pain has been evaluated in a randomized, placebo-controlled, inpatient rehabilitation setting [79, 80]. In one of these reports [79], patients receiving NMRT/MBST in addition to standardized inpatient rehabilitation demonstrated greater and more durable reductions in pain intensity than those treated with sham therapy. Improvements extended beyond pain reduction and included improved sleep quality as well as increased daily activities. Differences between groups became more pronounced during follow-up, suggesting delayed or cumulative biological effects that persisted beyond the immediate treatment period. Treatment acceptance was high, and no adverse effects were reported [79].

In the second report [80], the same trial was reported with a focus on VAS pain ratings and disability outcomes. Both the active NMRT/MBST and placebo groups showed reductions in pain shortly after treatment, but NMRT/MBST produced more consistent advantages in pain under stress and in several domains of functional disability. The NMRT/MBST group demonstrated more frequent improvements and fewer deteriorations in measures such as personal care and overall disability, whereas some domains—such as sleep and walking—improved similarly in both groups at follow-up [80].

Taken together, this study [79, 80] indicated that, while both NMRT/MBST and placebo groups show notable short-term improvements—consistent with the effects of standardized inpatient rehabilitation—MBST confers additional, domain-specific, and, in some cases, longer-lasting benefits, particularly for pain under stress, personal care, and overall disability. The two reports differ in emphasis, with the first [79] describing broader and more uniformly favorable NMRT/MBST-specific effects, whereas the second [80] documents a more differentiated pattern in which certain functional areas improved similarly across groups and others showed clear NMRT/MBST advantages.

These controlled findings are reinforced by a large observational dataset in which patients with degenerative spinal and rheumatic conditions reported sustained improvements in pain and functional disability across several months following NMRT/MBST [73]. Although observational in nature, these data provide insight into real-world responses across diverse care settings.

#### Lumbar disc herniation and radicular syndromes

The strongest controlled evidence for NMRT/MBST in spinal pathology comes from a double-blind, randomized trial in patients with lumbar disc herniation and associated radicular syndromes [81, 82]. In this study, NMRT/MBST produced clinically meaningful reductions in pain and significantly reduced sick-leave duration compared to placebo, indicating tangible functional benefit. These differences were most pronounced at early follow-up intervals and persisted over several months.

MRI demonstrated improvement in disc morphology in both treatment and placebo groups, consistent with natural recovery processes, but no significant between-group differences were detected [82]. This dissociation between functional recovery and short-term structural imaging changes suggests that NMRT/MBST primarily influences inflammatory, metabolic, and neurophysiological aspects of radicular pathology rather than directly accelerating disc resorption. The consistent reduction in sick-leave days [81] underscores the practical relevance of these effects.

#### Osteoporosis

Two clinical investigations provided insight into the potential role of NMRT/MBST in osteoporosis [83, 84]. In a prospective densitometric study, patients demonstrated statistically significant increases in bone mineral density at the lumbar spine and hip 1 year after treatment, accompanied by favorable changes in biochemical markers of bone formation [83], suggesting a shift toward anabolic bone metabolism. A larger retrospective cohort study monitored patients for several years after NMRT/MBST treatment and documented multiple episodes of severe trauma that resulted in substantial soft-tissue injury but no fractures [84]. The unexpectedly low fracture incidence during long-term follow-up suggests that NMRT/MBST may enhance bone quality or structural resilience in ways not fully captured by standard densitometric measures. Experimental evidence of enhanced osteoblast proliferation [41] and improved cellular energy homeostasis [43] provides a biologically plausible framework for these clinical observations.

#### Veterinary evidence

Veterinary investigations provide an additional perspective that is largely unaffected by placebo effects. A long-term case report described sustained functional improvement in a dog with severe hip OA following repeated annual cycles of NMRT/MBST, with mobility maintained for nearly a decade [85]. More rigorously, a double-blind controlled trial in dogs with elbow OA employed objective gait analysis and demonstrated measurable improvements in locomotor parameters after NMRT/MBST treatment [86]. Similarly, a randomized, double-blind, placebo-controlled veterinary trial in dogs with OA showed that NMRT/MBST produced significantly greater improvements in gait symmetry than placebo at 3 months post-treatment, accompanied by directional advantages in lameness scores and overall clinical response [87]. These between-group differences diminished by 6 months, and no sustained effects were observed thereafter. Collectively, these findings support both the biological efficacy of NMRT/MBST and the feasibility of blinded evaluation in nonhuman models.

#### Safety and tolerability

Across all clinical and veterinary investigations, NMRT/MBST has been consistently reported as safe and well tolerated. No treatment-emergent adverse events, therapy-related complications, discontinuations from intolerance, or delayed negative outcomes have been described. This favorable safety profile is consistent across repeated treatment cycles, long-term follow-up, and diverse patient populations.

### Cellular and molecular responses to NMRT/MBST

#### Proliferation and viability of musculoskeletal cells

NMRT/MBST promotes proliferation of human chondrocytes, osteoblasts, and fibroblasts without inducing cytotoxicity or apoptosis. Early investigations showed marked increases in cell numbers following intermittent exposure, with viability assays confirming preserved membrane integrity and metabolic activity [41]. Subsequent studies confirmed that NMRT/MBST supports regulated cell growth and preserves chondrocyte homeostasis under inflammatory or stress conditions [42–44], indicating stimulation of physiological rather than pathological proliferation. Importantly, not all musculoskeletal cell types responded with increased proliferation: in human dermal fibroblasts, NMRT/MBST did not alter proliferation or viability but instead modulated extracellular matrix (ECM) composition and collagen organization, indicating cell-type–specific and nonmitogenic regulatory effects [45]. These effects mirror those observed with PBMT/NILT, in which connective-tissue cells exhibit increased proliferation and survival under metabolic or inflammatory stress [55, 63]. The consistency across modalities supports the interpretation that NMRT/MBST activates conserved cellular repair and maintenance programs.

#### Anti-inflammatory and immunomodulatory effects

NMRT/MBST exerts pronounced anti-inflammatory effects in musculoskeletal cells exposed to inflammatory stimuli. In osteoarthritic chondrocytes, NMRT/MBST suppressed activation of key inflammatory transcription factors and reduced expression of matrix-degrading enzymes central to cartilage destruction [42–44]. At the same time, it preserved or restored anabolic regulators essential for ECM maintenance [42–44]. These coordinated effects shift the cellular environment from catabolic degradation toward tissue preservation. Comparable anti-inflammatory profiles are well established for PBMT/NILT [40, 55, 88], suggesting that NMRT/MBST engages shared inflammatory control pathways that are central to degenerative musculoskeletal disease.

#### Mitochondrial function, metabolism, and redox balance

NMRT/MBST improves mitochondrial function and cellular energy homeostasis, characterized by increased ATP availability, normalization of redox balance, reduced reliance on glycolysis, and stabilization of mitochondrial respiration [43, 44, 52–54]. NMRT/MBST normalizes pathological redox imbalance by reducing excessive NADH levels and restoring a healthier NAD^+^/NADH ratio [44]. This redox correction supported efficient electron transport [52, 53], stabilized mitochondrial respiration [54], and enhanced cellular oxidative homeostasis [44, 54]. Under hypoxic or inflammatory conditions, NMRT/MBST prevented maladaptive metabolic responses and normalized hypoxia-related signaling pathways [43, 44, 51–54, 61]. These metabolic effects closely parallel those induced by PBMT/NILT [55, 57, 89] and provide a mechanistic explanation for improved tissue resilience, reduced fatigue, and enhanced regenerative capacity observed clinically.

#### Chondrogenic, osteogenic, and matrix-regulatory effects

In chondrocytes, NMRT/MBST suppressed catabolic gene expression while restoring growth-factor signaling pathways and preserving key regulators of ECM synthesis [42, 44]. In osteoblasts, NMRT/MBST induced a marked increase in cell proliferation while preserving viability, consistent with stimulation of bone-forming processes [41]. These findings align closely with PBMT/NILT research demonstrating enhanced collagen synthesis, proteoglycan production, osteoblast differentiation, and mineralization [63, 89, 90]. The convergence of these effects provides a biological basis for clinical observations of symptom improvement in OA and increased bone density in osteoporosis.

#### Neurotrophic and neuroregenerative effects

In neuronal models, NMRT/MBST promoted neurite outgrowth and enhanced Schwann-cell–mediated neuro-supportive signaling, resulting in improved neuronal growth and metabolic support [52, 53]. These findings, which are also known for PBMT/NILT [58, 91], are particularly relevant for radicular pain syndromes and nerve irritation, where metabolic stress and inflammation impair neural function. Although no clinical applications to primary neurological disorders have been published, the experimental data suggest broader neurobiological relevance.

#### Epigenetic and circadian regulation

Beyond immediate signaling pathways, NMRT/MBST modulates gene regulation at the epigenetic level. It restored dysregulated microRNAs (miRNAs) involved in inflammation and matrix regeneration, altered histone-modifying enzyme activity such as HDAC4, and reshaped expression patterns of core circadian clock genes across several model systems [44, 51, 54, 61, 62]. Collectively, these epigenetic, metabolic, and circadian alterations, which align closely with PBMT/NILT research [92–94], indicate that NMRT/MBST may impart more lasting adjustments to cellular homeostasis, thereby modifying how cells respond to metabolic or inflammatory stressors over time [43, 44, 51, 54, 61, 62].

NMRT/MBST and PBMT/NILT converge mechanistically on cryptochrome-associated signaling but do so through fundamentally distinct biophysical entry points with different consequences for cellular timing and redox control. PBMT/NILT with 810 nm near-infrared light directly triggered ubiquitination-dependent degradation of the circadian clock protein cryptochrome 1 (CRY1), rapidly lowering nuclear CRY1 levels and derepressing downstream osteogenic pathways [93]. On the other hand, NMRT/MBST operates primarily through magnetic-field–sensitive radical-pair processes that influence cryptochrome indirectly via changes in intracellular reactive oxygen species (ROS) partitioning [62]. This magnetic-field–driven modulation alters CRY1, CRY2, and CLOCK1 steady-state levels in a distinctly nonlinear, window-dependent fashion, without inducing proteasomal degradation or direct photoreceptor activation [61]. Related studies demonstrated that the resulting shifts in redox balance feed into HIF-1α expression, glycolytic flux, mitochondrial respiration, and time-of-day–dependent “on/off” effects on Per2-driven circadian oscillations [54, 62]. Moreover, cross-species comparisons indicate that mammalian CRY-linked radical-pair pathways are considerably more responsive to NMRT/MBST than those of zebrafish [51], pointing to organism-specific susceptibilities in cryptochrome-mediated magnetic sensing.

Together, these findings suggest that while both NMRT/MBST and PBMT/NILT act on cryptochrome-centered regulatory networks, NMRT/MBST does so by reconfiguring the redox-sensitive circadian and hypoxia-responsive machinery rather than by inducing direct CRY1 turnover. This distinction highlights NMRT/MBST as a modality capable of influencing the temporal organization of cellular metabolism and stress responsiveness through persistent adjustments in ROS signaling, HIF-1α dynamics, and cryptochrome-dependent gene regulation—mechanisms that may contribute to its reported therapeutic benefits in inflammatory, degenerative, and ischemia-related conditions.

#### Radical-pair mechanisms and redox signaling

Mechanistic studies indicate that NMRT/MBST operates through redox-sensitive radical-pair processes consistent with cryptochrome-mediated magnetosensing [54, 61, 62]. NMRT/MBST reshaped intracellular ROS signaling in a tightly regulated manner, and these effects critically depend on intact radical intermediates rather than nonspecific oxidative stress [54]. This mechanism closely parallels the controlled ROS signaling induced by PBMT/NILT [40, 95], providing a unifying framework to understand how distinct physical stimuli converge on shared biological outcomes.

### Penetration depth and therapeutic reach

A defining distinction between NMRT/MBST and PBMT/NILT (or optical therapies in general) lies in tissue penetration. Near-infrared light is limited by absorption and scattering, restricting its effective range to superficial structures [17–19]. NMRT/MBST, by contrast, employs magnetic and low-frequency RF fields that are not meaningfully attenuated by biological tissue [33–35]. As a result, entire joints, spinal segments, and deep skeletal structures can be exposed uniformly, regardless of anatomical depth or complexity.

This unrestricted penetration provides a coherent explanation for the effectiveness of NMRT/MBST in conditions dominated by deep tissue pathology, including radicular syndromes, deep-joint OA, and osteoporosis. It also positions NMRT/MBST as a complementary modality capable of extending PBMT/NILT-like biological effects to regions beyond the reach of light-based therapies.

## Discussion

### A shifting therapeutic landscape and the role of deep biophysical treatments

The management of chronic musculoskeletal and skeletal disorders is undergoing a conceptual evolution. Historically, treatment pathways have relied heavily on pharmacological symptom control, mechanical unloading, physical therapy, and, when conservative options fail, surgical intervention [5–7]. Yet millions of patients live with conditions that do not progress to surgical indications, remain symptomatic despite medication, or suffer adverse effects from long-term drug use. Against this backdrop, noninvasive biophysical therapies have emerged as compelling adjuncts [13–16]. Among them, PBMT/NILT has gained meaningful clinical and mechanistic support [15, 38–40]. However, the inherent depth limitation of PBMT/NILT means that its most robust effects are restricted to surface tissues and shallow musculoskeletal structures [17–19].

This constraint has left a substantial gap: the need for a noninvasive therapy capable of safely delivering meaningful biological modulation to deep tissues such as intervertebral discs, subchondral bone, trabecular structures, and large weight-bearing joints. NMRT/MBST enters this landscape as a modality designed explicitly to address this unmet need. Its mode of action, based on low-intensity magnetic fields and RF signals configured to induce magnetic-resonance interactions, differs fundamentally from optical and mechanical therapies. Crucially, the energy delivery of NMRT/MBST is not attenuated by tissue depth or composition [33–35], enabling homogeneous exposure of entire anatomical regions irrespective of size, shape, or depth. This distinctive property provides not only a technical advantage but also a theoretical basis for why NMRT/MBST may exert clinically meaningful effects in conditions where surface-limited therapies have shown inconsistent benefit.

Preclinical translational data further reinforce this perspective. In a blinded, randomized ACL-transection rabbit model, NMRT/MBST initiated at an early post-injury stage produced significantly lower macroscopic OA severity compared with sham treatment, driven by fewer fibrillations, reduced osteophyte formation, and less joint effusion. When initiated later, these macroscopic benefits were absent, and histologic Mankin scores were unchanged in both treatment windows [77]. This timing-dependent effect suggests that NMRT/MBST acts most effectively during the early inflammatory and metabolic phases of joint degeneration before irreversible matrix breakdown develops [77].

### Broadening the biological model for noninvasive regenerative therapies

Modern biophysical therapies (especially PBMT/NILT) can be conceptualized as modulators of cellular bioenergetics, inflammation, and tissue homeostasis [40, 55, 57]. Over the last 2 decades, PBMT/NILT has established a comprehensive mechanistic framework describing how controlled photonic energy influences mitochondrial function, redox biology, inflammatory transcription factors, and growth-factor signaling [38–40, 55, 57]. Interestingly, these pathways represent precisely the domains in which NMRT/MBST exerts experimentally verified effects [41–45, 52–54, 61, 62]—even though the initiating stimulus is entirely different.

This convergence invites a broader theoretical model in which noninvasive therapies modulate shared intracellular systems through distinct external inputs. Under this model, PBMT/NILT and NMRT/MBST are not competing or contradictory approaches but rather parallel routes into the same regulatory architecture of the cell. If PBMT/NILT represents a light-driven mechanism to enhance mitochondrial and metabolic equilibrium, NMRT/MBST can be conceptualized as a resonance-driven mechanism capable of producing comparable downstream outcomes without optical penetration restrictions.

Such a model helps explain the remarkable consistency observed across mechanistic domains while also recognizing the unique advantages and boundaries of each therapy.

### Mechanistic convergence: parallels between NMRT/MBST and PBMT/NILT

Table 3 offers a consolidated comparison of the presently established biological effects of NMRT/MBST and PBMT/NILT, summarizing points of mechanistic convergence and divergence across both modalities.

**Table 3:** Consolidated comparison of the presently established biological effects of NMRT/MBST and PBMT/NILT, summarizing points of mechanistic convergence and divergence across both modalities.

Effect/outcome	NMRT/MBST	PBMT/NILT
Cell proliferation and viability	Increases proliferation of chondrocytes and osteoblasts (e.g. +270% versus control by day 15 *in vitro*) [41]. No viability loss or apoptosis seen; maintains healthy cell counts (no cytotoxic effect in culture) [41, 42].	Increases proliferation of fibroblasts, osteoblasts, chondrocytes—enhancing tissue growth and repair [46–48]. Does not harm viability; can improve cell survival under stress (e.g. better cell integrity in burn-injured tissue) [55, 56].
Anti-inflammatory effects	Down-regulates NF-κB under inflammatory challenge (IL-1β–stimulated cells) [42, 43]. Reverses inflammatory gene changes: inhibits IL-1β–induced MMP-3, MMP-13, and Runx1 while restoring HIF-1α [42–44].	Suppresses inflammatory mediators: lowers NF-κB activity and pro-inflammatory cytokines (IL-1β, TNF-α, IL-6) [40, 49, 50]. Reduces iNOS/COX-2 expression and inflammatory cell infiltration [40, 88].
Mitochondrial function and metabolism	Recharges metabolism toward aerobic respiration: reduces glycolysis (↓lactate, ↓ECAR) and increases cellular energy charge [43, 44]. Preserves/improves ATP levels under stress (e.g. inflammatory hypoxia); creates a more reduced intracellular redox state (↓NAD^+^/NADH ratio, ↑NADH utilization) [44, 52, 54]	Boosts mitochondrial ATP production and oxidative metabolism (↑ATP/ADP, ↓lactate) [38, 39, 55, 57]. Stabilizes mitochondria: maintains membrane potential, prevents cytochrome c release and excess ROS [55, 57].
Chondrogenic and osteogenic stimulation	Stimulates cartilage and bone cell proliferation for tissue regeneration [41, 42, 44]. Improves cartilage homeostasis: counteracts catabolic enzymes (↓MMPs) and supports anabolic factors (↑IGF, maintains HIF-1α) [42–44].	Stimulates osteoblast and chondrocyte activity, leading to increased matrix formation [46, 47, 89, 90]. Accelerates wound and bone-defect healing via growth-factor modulation (BMPs, TGF-β) [63, 89].
Neuronal regeneration and neuroprotection	Promotes nerve regeneration via growth-factor release: NMRT-stimulated glial cells secrete more β-NGF, enhancing neurite outgrowth [52, 53]. Enhances neurite extension and branching in dorsal root ganglion neurons *ex vivo*; suggests potential neuroprotective effects [52].	Reduces neuroinflammation and oxidative stress; neuroprotective in Alzheimer’s and Parkinson’s models [138]. Increases neurogenesis and synaptogenesis; elevates neurotrophic factors and cerebral blood flow [58, 91].

#### Shared modulation of mitochondrial bioenergetics

Both NMRT/MBST and PBMT/NILT significantly increase mitochondrial efficiency, although by different physical mechanisms [38, 39, 43, 44, 52–55, 57, 62]. PBMT/NILT operates by photon absorption in red and near-infrared wavelengths, particularly in cytochrome c oxidase [38, 39]. This interaction enhances electron transport, increases ATP production, and improves the membrane potential of mitochondria [38, 39, 55, 57]. NMRT/MBST, by contrast, appears to influence electron transport and energy homeostasis through resonance-dependent interactions with radical pairs and magnetosensitive flavoproteins, particularly cryptochrome [54, 61, 62].

Despite these fundamentally different initiating events, the cellular consequences show overlap: improved ATP availability, reduced adenosine diphosphate (ADP)/ATP ratios, more stable oxidative phosphorylation, and reduced reliance on glycolysis [38, 39, 43, 44, 54, 55, 57, 62]. These effects are pivotal in tissues with high metabolic demands and limited vascularity—such as cartilage, intervertebral discs, or regions of trabecular bone—where metabolic resilience strongly influences pain, function, and degeneration [96–98].

#### Parallel effects on redox signaling and controlled reactive oxygen species dynamics

Modern research increasingly recognizes that therapeutic modulation of ROS must be neither excessive nor suppressive but optimally balanced to activate adaptive cellular pathways [99–101]. PBMT/NILT induces a brief, controlled burst of mitochondrial ROS, which serves as a signaling trigger for antioxidant upregulation (e.g. Nrf2 activation), growth-factor release, and reduced chronic inflammation [38–40, 55, 57]. NMRT/MBST appears to mirror this dynamic, producing shifts in peroxide and superoxide compartmentalization via radical-pair mechanisms. These changes influence redox-sensitive transcription factors, including those governing inflammation, cell survival, and matrix turnover [42–44, 54, 61, 62]. This parallel suggests that both NMRT/MBST and PBMT/NILT harness low-level ROS as a signaling intermediate rather than as a destructive force, activating restorative and defensive programs that outlast the initial stimulus.

#### Inflammatory control and transcriptional modulation

In a wide range of cell types—chondrocytes, osteoblasts, fibroblasts, and neural cells—PBMT/NILT downregulates NF-κB activity, reduces inflammatory cytokine expression, and rebalances the inflammatory milieu toward resolution [40, 55, 88]. NMRT/MBST demonstrates nearly identical anti-inflammatory signatures, including suppression of catabolic cytokine-driven genes, preservation of anabolic and hypoxia-adaptive transcription factors such as HIF-1α, and stabilization of ECM metabolism [42–44, 51, 52, 54, 61]. These responses help explain clinical findings in OA and radiculopathy, where inflammation—not structural deformation alone—drives pain and dysfunction [102–106].

#### Stimulation of regeneration in musculoskeletal tissues

PBMT/NILT stimulates collagen synthesis, proteoglycan production, fibroblast proliferation, osteoblast differentiation, and neurite extension [58, 63, 89–91]. NMRT/MBST activates analogous pathways: it enhances osteoblast and chondrocyte proliferation, preserves matrix-production capacity under inflammatory stress, and stimulates neurite extension via neurotrophic signaling [41–44, 52, 53]. This implies that NMRT/MBST and PBMT/NILT activate comparable regenerative programs, but NMRT/MBST can do so in deep tissues where light cannot effectively penetrate.

#### Epigenetic and circadian regulation

Both NMRT/MBST and PBMT/NILT influence epigenetic modifiers such as histone acetylation, HDAC regulation, and miRNA expression [44, 51, 54, 61, 62, 92–94]. They also modulate circadian genes [44, 51, 54, 61, 62, 92–94]—an emerging area in musculoskeletal biology [107–111]—implicating deeper regulatory systems that influence metabolism, repair, and inflammation across time. NMRT/MBST has demonstrated changes in the oscillatory amplitude of clock genes [54, 62], aligning with PBMT/NILT findings that link circadian regulation to enhanced tissue recovery [92–94]. Together, these parallels reinforce the conceptualization of NMRT/MBST as a deep-tissue analog of PBMT/NILT, accessing the same intracellular regulatory pathways via a resonance-based physical mechanism.

The timing-sensitive effects observed in the ACL-transection rabbit model [77] support this mechanistic interpretation. NMRT/MBST produced macroscopic improvements only when delivered in an early post-injury window, paralleling *in vitro* findings that the modality most effectively modulates inflammatory, redox, and metabolic stress responses before irreversible matrix deterioration occurs. The absence of histologic differences at later initiation points underscores that NMRT/MBST influences upstream biological processes that precede overt structural degeneration [77].

### Clinical interpretation: understanding the variability across indications

The expanded clinical evidence reveals a nuanced picture: certain indications show strong, consistent improvements [73, 78, 79, 81, 82, 85, 86], while others present mixed results depending on trial design, outcome measurements, and biological considerations [70–72].

#### Osteoarthritis: structural versus functional outcomes

Finger-joint OA has shown the clearest controlled evidence of superiority over placebo [78]. This success may relate to the small joint environment, where inflammatory and metabolic processes influence symptoms more directly than large-joint biomechanical load [112]. In knee OA, structural heterogeneity, high placebo responsiveness, and slower cartilage turnover complicate short-term detection of treatment effects [74–76, 113, 114]. Nevertheless, real-world observational data and long-term follow-up suggest that NMRT/MBST may exert meaningful influence on symptom trajectories [72, 73].

#### Spinal pain and radiculopathy: biological versus structural correlates

In radicular syndromes, the dissociation between early functional improvements and unchanged short-term imaging aligns with contemporary understanding of spine pain [64, 115]. Symptoms often arise from neuroinflammation, metabolic dysfunction within nerve tissues, and microenvironmental stress, rather than from static structural encroachment [116, 117]. NMRT/MBST’s anti-inflammatory and neurotrophic effects thus offer a plausible mechanism for these clinical improvements.

#### Osteoporosis: beyond bone mineral density

In addition to the observed increases in bone mineral density [83], the relevance of NMRT/MBST for osteoporosis is underscored by anecdotal reports of higher trauma tolerance without fracture following treatment [84]. Bone mineral density alone does not reliably predict fracture risk [118, 119], and the demonstrated biological effects of NMRT/MBST on osteoblast proliferation, cellular energy metabolism, and redox regulation [41–44, 52–54, 61, 62] position this modality closer in concept to established anti-fracture medications [120]. If validated in further studies, NMRT/MBST could represent a rare nonpharmacological intervention with the potential to enhance both bone mass and bone resilience.

#### Veterinary medicine: independent confirmation

Evidence from veterinary models—particularly objective gait analysis in controlled studies—provides additional support that NMRT elicits biological effects independent of expectation or placebo mechanisms [86, 87]. The multiyear effectiveness observed in long-term veterinary cases further highlights NMRT’s potential durability.

### Theoretical implications: toward a unified model of deep bioenergetic modulation

The mechanistic parallels between NMRT/MBST and PBMT/NILT, combined with the depth-independent penetration of NMRT/MBST, support the following unified conceptual framework: noninvasive biophysical therapies exert their therapeutic effects by modulating mitochondrial, redox, inflammatory, and regenerative networks. PBMT/NILT operates through photon–chromophore interactions; NMRT/MBST operates through magnetic-resonance interactions with radical pairs and magnetosensitive proteins. The downstream consequences converge.

Under this model, NMRT/MBST is not merely an electromagnetic analog of PBMT/NILT; it is an extension of the same biological logic into the deep anatomical domain. This framing has several implications. First, deep musculoskeletal tissues—including discs, subchondral bone, deep-joint cartilage, and central joint spaces—may be amenable to noninvasive bioenergetic therapy for the first time. Second, combined therapy strategies may become possible—PBMT/NILT for superficial tissues; NMRT/MBST for deep structures—harmonizing both modalities in a comprehensive regenerative approach. Third, mechanistic biomarkers may help personalize therapy. Redox state, mitochondrial profile, inflammatory signatures, and circadian markers could guide optimal dosing and candidate selection [40, 55, 88, 121–127].

### Current limitations

While the integrated evidence is compelling, several limitations temper final conclusions. First, controlled trials employing NMRT/MBST remain few, with modest sample sizes in several indications. Second, structural imaging endpoints have not consistently mirrored symptomatic improvements, reflecting the complex relationship between imaging and clinical outcomes. Third, optimal treatment parameters—frequency, field strength, duration, and cumulative dose—remain to be rigorously defined. Fourth, comparative studies directly evaluating NMRT/MBST versus PBMT/NILT across tissues of differing depth have not yet been performed. Fifth, the impact on bone quality, circadian regulation, and epigenetic dynamics requires dedicated longitudinal investigation. Priorities for future research include larger randomized trials, mechanistically informed biomarkers, head-to-head comparisons with PBMT/NILT, and exploration of synergistic combination protocols.

### Broader biophysical horizons: what PBMT/NILT mechanisms reveal about the untapped mechanistic landscape of NMRT/MBST in musculoskeletal disorders

While the present review summarizes the molecular and cellular mechanisms of NMRT/MBST that are currently supported by experimental data, this likely represents only a partial view of the modality’s broader biophysical potential. The historical trajectory of PBMT/NILT offers a relevant perspective: decades of research have gradually revealed multiple mechanistic pathways far beyond the initially hypothesized mitochondrial chromophore activation. This evolution in understanding underscores how emerging biophysical therapies often begin with a narrow mechanistic model that later expands as more diverse cellular and neurophysiological effects are uncovered. Importantly, none of the mechanistic or clinical studies discussed below investigated NMRT/MBST. However, they illustrate the range of biological actions that become conceivable if NMRT/MBST were to share—even partially—the nonthermal, low-energy biophysical mechanisms described for PBMT/NILT, without being limited by the shallow penetration depth of PBMT/NILT.

Across musculoskeletal medicine, several well-characterized PBMT/NILT-based effects illustrate this potential. Deep nociceptor modulation demonstrated in early neurophysiological work [128] and anti-inflammatory suppression of prostaglandin E_2_ in human tendinopathy [129] highlight how PBMT/NILT can influence both pain-processing networks and local inflammatory mediators. These are mechanistic domains that are of broad relevance for NMRT/MBST, which aims to modulate musculoskeletal tissue physiology and symptom generation throughout entire anatomical volumes rather than only superficial layers. Likewise, the attenuation of trauma-induced NF-κB and inducible nitric oxide synthase (iNOS) activity and prevention of fibrosis in structurally injured skeletal muscle [130] expands the concept of PBMT/NILT from symptomatic relief to true modulation of tissue healing. If NMRT/MBST were capable of producing analogous anti-nociceptive, anti-inflammatory, or anti-fibrotic responses in injured muscles, but at far greater tissue depth, it could synergize with ESWT in ways surpassing the regeneration observed in animal models of structural muscle injury [131] and potentially improve clinical outcomes beyond what was achieved in ESWT-based protocols for functional and structural muscle injuries in professional athletes [132, 133].

Two additional clinical domains further illustrate this horizon. In controlled studies on myofascial pain syndrome, PBMT/NILT and dry needling reduced pain and improved pressure thresholds [134, 135], yet both interventions are constrained by the inaccessibility of deep or anatomically hazardous trigger points, most notably the psoas major muscle, whose myofascial trigger points are now recognized as a major contributor to low-back, hip, and groin pain and a key driver of lumbopelvic dysfunction [136], while also being exceptionally difficult to access safely given its deep location and proximity to visceral and neurovascular structures. A noninvasive whole-tissue modality such as NMRT/MBST, not restricted by the millimeter-range penetration of optical wavelengths, could theoretically modulate deep myofascial dysfunction in regions entirely beyond the reach of PBMT/NILT. Similarly, in whiplash-associated disorders, high-power laser therapy has demonstrated meaningful reductions in pain and faster return to work compared to conventional rehabilitation [137], but again only within the constrained envelope of optical penetration. If NMRT/MBST shares any of the same downstream biological mechanisms, yet acts volumetrically across the cervical musculoligamentous complex, it could offer a broader therapeutic impact in patients whose symptoms arise from tissues situated beyond the effective depth of PBMT/NILT delivery.

Together, these mechanistic and clinical examples demonstrate how PBMT/NILT has progressively uncovered multiple layers of nonthermal biological action (nociceptive, inflammatory, metabolic, and transcriptional) yet remains fundamentally limited by its shallow penetration. NMRT/MBST, if it engages comparable pathways at clinically meaningful depths, may ultimately enable therapeutic effects in musculoskeletal disorders that exceed both the reach and the scope of PBMT/NILT-based interventions.

### Broader biophysical horizons: exploring PBMT/NILT-informed mechanistic pathways potentially accessible to NMRT/MBST in neurodegenerative and neuroinflammatory diseases

In the context of neurodegenerative diseases, PBMT/NILT has long been proposed to counteract progressive neuronal dysfunction through mechanisms such as mitochondrial stabilization, improved oxidative balance, modulation of neuroinflammation, and support of synaptic maintenance and plasticity [138]. These mechanistic hypotheses generated substantial expectations, especially the idea that low-energy, nonthermal stimulation might not only slow degenerative decline but potentially enhance endogenous repair pathways within vulnerable neuronal populations. However, the translation of these concepts into clinically meaningful outcomes has been consistently limited by the fundamental physical constraints of light penetration: even at near-infrared wavelengths, even superficial cortical regions cannot be reached at therapeutic intensities. This depth barrier has been highlighted repeatedly, including in recent experimental work suggesting that externally applied photonic energy cannot produce measurable changes in cortical tissue in a reliable or physiologically significant manner [19]. These observations underscore a central limitation of PBMT/NILT: promising biochemical and cellular mechanisms exist, yet their practical application is constrained by tissue optics rather than biological potential.

Against this backdrop, NMRT/MBST represents a physically distinct class of biophysical stimulation that is not subject to optical attenuation and thus offers an opportunity to test whether deeper neural structures might respond to NMRT/MBST in ways conceptually analogous to those proposed for PBMT/NILT. Early *in vitro* observations provide tentative but encouraging indications. In experiments on peripheral neuronal and glial cultures, NMRT/MBST exposure enhanced neurite outgrowth, promoted neuronal survival, and influenced glial support functions under controlled conditions, including increased release of neurotrophic factors that support regeneration [52]. In additional studies, such stimulation modulated the secretory activity of supportive glial cells, elevating levels of key neurotrophins without inducing unwanted sensitization responses in nociceptive neurons [53]. Although these effects remain confined to peripheral and *in vitro* preparations, they illustrate that NMRT/MBST can influence cellular programs that are highly relevant for neurodegenerative disorders, especially as they involve axonal vulnerability, impaired trophic support, and glial dysfunction.

A further illustration of this PBMT/NILT-derived blueprint is provided by multiple sclerosis (MS). In MS, recent experimental and clinical work has shown that PBMT/NILT can modulate several pathophysiological hallmarks of the disease, including suppression of pro-inflammatory cytokines, attenuation of microglial and astroglial activation, reduction of oxidative and nitrosative stress, and partial preservation of oligodendrocyte lineage cells, while improving motor, sensory, and functional scores in patients and experimental autoimmune encephalitis (EAE) models alike [139]. Such findings might be especially relevant during the progressive disease stages where treatment options are still limited. These findings also reinforce that low-energy biophysical stimulation can engage neuroimmune, mitochondrial, and anti-apoptotic pathways highly relevant to demyelinating disease, yet PBMT/NILT remains physically unable to deliver these effects. If NMRT/MBST could access even a subset of these mechanisms at depth, particularly those related to glial modulation, redox stabilization, and trophic support, it could theoretically target deep periventricular and spinal white-matter regions that remain inaccessible to photonic approaches. Although entirely untested at present, MS thus represents a prototypical condition in which PBMT/NILT-identified mechanisms intersect with the depth-independent delivery profile of NMRT/MBST, providing a concrete rationale for future mechanistic exploration.

At the same time, it is essential to recognize that the mechanistic understanding of NMRT/MBST effects on the nervous system remains at a very early stage. No data currently address central neurons, neural networks, microglial states, or interactions within the neurovascular unit. The limited evidence available is restricted to peripheral glia and sensory neurons [52, 53] and therefore does not yet permit conclusions about potential effects within the brain or spinal cord. Nevertheless, the combination of (i) the mechanistic blueprint provided by PBMT/NILT research; (ii) the absence of a penetration-depth barrier for NMRT/MBST; and (iii) the preliminary *in vitro* evidence for neurotrophic and pro-regenerative actions together offers a scientifically grounded rationale for further exploration. Should future studies reveal that NMRT/MBST can safely modulate neural tissue at meaningful depths, this modality could potentially address therapeutic targets in neurodegenerative and neuroinflammatory conditions that have remained inaccessible to light-based approaches. Such a trajectory remains speculative at present but represents a worthwhile and increasingly actionable avenue for rigorous mechanistic and translational research.

### Conventional pulsed electromagnetic field therapy as a comparator: physical proximity does not imply mechanistic equivalence

Conventional PEMFT represents an important comparator when positioning NMRT/MBST within the broader landscape of nonthermal, nonmechanical biophysical therapies. At first sight, PEMFT may appear more closely related to NMRT/MBST than PBMT/NILT because both employ magnetic fields, whereas PBMT/NILT is based on optical radiation. However, similarity at the level of the externally applied physical stimulus should not be equated with either the primary tissue interaction or the subsequent biological mechanism. Conventional PEMFT employs time-varying magnetic fields that generate induced electric fields and currents in conductive tissue, whereas NMRT/MBST combines a static magnetic field with controlled sweep fields and low-frequency RF excitation under nuclear magnetic resonance conditions. PBMT/NILT differs physically from both, relying on photon absorption by endogenous photoacceptors. Thus, all three modalities are noninvasive and nonthermal under therapeutic conditions, but they introduce physical energy into biological systems through fundamentally different routes.

These physical distinctions also produce different constraints on target exposure. PBMT/NILT has the clearest intrinsic depth limitation because photon fluence decreases markedly through absorption and scattering in intervening tissue. This becomes particularly relevant for deep cartilage, subchondral bone, intervertebral discs, and central neural structures [17–19]. Conventional PEMFT avoids this optical attenuation and can reach deeper tissues, but this should not be interpreted as depth-independent or spatially uniform biological exposure. Magnetic-field strength and, particularly, the induced electric field depend on coil geometry, distance, orientation, waveform, and rate of field change. Consequently, conventional PEMFT replaces the photon-penetration problem of PBMT/NILT with a spatial electromagnetic-dosimetry problem [22–25]. NMRT/MBST differs again: its static, sweep, and RF fields are configured to establish resonance conditions throughout a defined treatment volume and are not progressively depleted by absorption or scattering in intervening biological tissue [33–35]. These physical differences are important for device characterization, dosimetry, and safety, but physical similarity alone does not determine which modality represents the most informative biological comparator.

At the mechanistic level, the distinction becomes more consequential. Conventional PEMFT has accumulated substantial experimental evidence for membrane-associated signal transduction [140–142], modulation of Ca^2+^-dependent pathways [143–145], and, particularly, A_2_A and A_3_ adenosine-receptor signaling [146–148], with subsequent effects on cAMP [141, 146, 147], kinases [149–151], NF-κB [145, 147, 152], vascular responses [147, 150, 153], extracellular-matrix regulation [149, 150, 154], and cellular differentiation [149, 150, 155]. Mitochondrial and redox changes have also been reported [143, 144, 152], but they are more commonly positioned downstream within this signaling architecture than as the primary biological transducer. By contrast, PBMT/NILT has developed the broadest mechanistic framework around mitochondrial photobiology, bioenergetics, transient redox signaling, nitric oxide, and subsequent transcriptional regulation [38–40, 55, 57]. Importantly, the NMRT/MBST evidence summarized in the present review shows unexpectedly extensive convergence with this PBMT/NILT downstream biology despite fundamentally different initiating physics: both modalities modulate mitochondrial energy metabolism, redox homeostasis, Ca^2+^ signaling, inflammatory pathways, gene and epigenetic regulation, extracellular-matrix turnover, differentiation, hypoxia adaptation, circadian regulation, and neuroregenerative signaling [41–45, 51–54, 61, 62]. The mechanistic rationale for using PBMT/NILT as the principal biological comparator in this review therefore rests not on physical similarity but on this unusually broad convergence of experimentally demonstrated downstream cellular responses.

PEMFT nevertheless provides important independent support for the broader concept that weak, nonthermal electromagnetic exposure can produce biologically and clinically meaningful effects. Its most established historical application is skeletal regeneration, particularly delayed fracture healing and nonunion. Subsequent research has extended to OA, osteoporosis, spinal disorders, wound healing, tendinopathies, and neurological applications [22–25, 156–158]. However, “PEMF” encompasses an exceptionally heterogeneous signal space, including substantial differences in field strength, frequency, waveform, pulse structure, applicator geometry, and treatment schedule [159–161]. Consequently, evidence obtained with one PEMF device cannot automatically be generalized to another, and still less can it be transferred directly to NMRT/MBST merely because both modalities employ magnetic fields. This distinction is especially relevant when comparing conventional induced-electric-field mechanisms with the resonance-based exposure paradigm of NMRT/MBST.

From a clinical perspective, the three modalities should therefore not be ranked according to physical resemblance but evaluated indication by indication. Differences in the volume of published evidence partly reflect historical adoption: PEMFT developed particularly strongly around bone healing [162–164], whereas PBMT/NILT became established across numerous superficial or optically accessible indications. Such differences do not necessarily imply biological exclusivity. Conversely, mechanistic evidence may occasionally suggest genuine differences in therapeutic suitability, emphasizing that shared downstream endpoints do not guarantee equivalent effects in every condition. For NMRT/MBST, the most informative comparator therefore depends on the level of analysis: PEMFT is the more obvious comparator in terms of the applied physical stimulus, whereas PBMT/NILT shows the more extensive experimentally demonstrated overlap in downstream molecular and cellular responses. Neither modality should be regarded as a mechanistic surrogate for NMRT/MBST, and ultimately only direct clinical evidence can establish patient benefit for a defined indication.

## Conclusion

NMRT/MBST emerges as a safe, biologically coherent and clinically promising noninvasive therapy capable of modulating deep-tissue physiology in ways previously accessible only to optical or invasive modalities. Across musculoskeletal conditions, including OA, chronic low back pain, radicular syndromes, and osteoporosis, studies report consistent signals of pain reduction, functional improvement, and, in selected contexts, structural or densitometric change, with an exceptionally favorable safety profile and durable benefits.

Mechanistically, NMRT/MBST engages a broad regulatory network encompassing mitochondrial bioenergetics, redox signaling, inflammatory control, anabolic cartilage and bone pathways, neurotrophic activation, and epigenetic and circadian modulation. These effects closely parallel those of near-infrared PBMT/NILT, yet NMRT/MBST uniquely delivers depth-independent stimulation, enabling access to tissues not reachable by light-based therapies.

Conventional PEMFT provides an additional but distinct comparator: although physically closer to NMRT/MBST through its use of magnetic fields, its predominantly membrane-, ion-, and receptor-associated mechanistic framework differs from the extensive downstream biological convergence demonstrated between NMRT/MBST and PBMT/NILT.

Together, the clinical evidence and mechanistic plausibility position NMRT/MBST as a meaningful addition to conservative musculoskeletal care. While current data are encouraging, larger randomized trials, optimized dosing studies, and mechanistic biomarker-driven research remain essential to fully define its therapeutic role and long-term impact.

## Supplementary Material

Integrated_Review_NMRT-MBST_for_BMB_Appendix_ldag045

## Data Availability

The data underlying this article are available in the article and in its online supplementary material.
