# Supplementary material for: Nuclear magnetic resonance therapy for deep-tissue biophysical stimulation: an integrated clinical and molecular analysis
Source: Br Med Bull. 2026 Nov 9;160(1):ldag045. doi: 10.1093/bmb/ldag045 (PMC13747023; doi:10.1093/bmb/ldag045)
Supplement: Supplementary file 1 [file integrated_review_nmrt-mbst_for_bmb_appendix_ldag045.docx]

**Nuclear magnetic resonance therapy for deep-tissue biophysical stimulation: an integrated clinical and molecular analysis**

Dalibor Krpan, Alireza Monajati, Aliya Yasmeen, Adam Benn, Ramy Atef Shenouda, Patrick R. Hof, Markus Kipp, Charles Mamisch, Anna J. Schreiner, Nicola Maffulli, Christoph Schmitz

**Appendix – Details of the Systematic Literature Search Performed on December 28, 2025**

**Table A1** | Search strategies: number of searches by search terms and databases used (as referenced in Table A2).

| **Search terms** | **PubMed** | **Embase/OVID** |
| --- | --- | --- |
| MBST | 1 | 8 |
| MBST therapy | 2 | 9 |
| NMRT | 3 | 10 |
| tNMR | 4 | 11 |
| nuclear magnetic resonance therapy | 5 | 12 |
| therapeutic nuclear magnetic resonance | 6 | 13 |
| molecular biophysical stimulation therapy | 7 | 14 |

**Table A2** | Detailed results of the systematic literature search.

Abbreviations: N_1_, consecutive study number; N_2_, consecutive number within the respective category; T, type of study (O, original study; R, review; C, conference abstract; N, not relevant); Search, search strategy in Table A1 in which the corresponding study was identified.

For illustration, the study by Gökşen et al. (consecutive study number 1; *Eur J Phys Rehabil Med* 2016;52(4):431–9) was identified using the search terms “nuclear magnetic resonance therapy” and “therapeutic nuclear magnetic resonance” in PubMed (Searches 5 and 6), and twice each with the same search terms in Embase/Ovid (Searches 12 and 13). The occurrence of duplicate hits in Embase/Ovid is attributable to the fact that the search was conducted across all available resource subsets within that database.

| **N_1_** | **N_2_** | **T** | **Reference** | **Search** |
| --- | --- | --- | --- | --- |
| 1 | 1 | O | Gökşen et al. Magnetic resonance therapy for knee osteoarthritis: a randomized, double blind placebo controlled trial. *Eur J Phys Rehabil Med* 2016;52(4):431-9. | 5, 6, 12 (2x), 13 (2x) |
| 2 | 2 | O | Huels et al. Treatment of the clinical symptoms of osteoarthritis in the elbow joints of dogs using nuclear magnetic resonance therapy: a randomized, double-blinded trial. *Front Vet Sci* 2020;7:500278. | 1, 2, 5, 8(2x), 9 (2x), 12 (2x) |
| 3 | 3 | O | Jansen et al. Effects of low-energy NMR on posttraumatic osteoarthritis: observations in a rabbit model. *Arch Orthop Trauma Surg* 2011;131(6):863-8. | 1, 2, 8 (2x), 12 |
| 4 | 4 | O | Krpan and Kullich. Nuclear magnetic resonance therapy (MBST) in the treatment of osteoporosis. Case report study. *Clin Cases Miner Bone Metab* 2017;14(2):235-8. | 1, 2, 3, 5, 6, 8, ,9, 10, 12, 13 |
| 5 | 5 | O | Krpan et al. Non-pharmacological treatment of osteoporosis with nuclear magnetic resonance therapy (NMR-therapy). Period Biol 2015;117(1):161-165. | 8, 9, 12 |
| 6 | 6 | O | Kullich et al. Additional outcome improvement in the rehabilitation of chronic low back pain after nuclear resonance therapy. *Rheumatologia* 2006;20(1):7-12. | 8, 9 |
| 7 | 7 | O | Kullich et al. One-year-survey with multicenter data of more than 4,500 patients with degenerative rheumatic diseases treated with therapeutic nuclear magnetic resonance. *J Back Musculoskelet Rehabil* 2013;26(1):93-104. | 6, 13 (2x) |
| 8 | 8 | O | Kullich et al. The effect of MBST-NuclearResonanceTherapy with a complex 3-dimensional electromagnetic nuclear resonance field on patients with low back pain. *J Back Musculoskelet Rehabil* 2006;19(2-3):79-87. | 8, 9 |
| 9 | 9 | O | Mann et al. Nuclear magnetic resonance treatment accelerates the regeneration of dorsal root ganglion neurons in vitro. *Front Cell Neurosci* 2022;16:859545. | 3, 5, 10 (2x), 12 (2x) |
| 10 | 10 | O | Mucha et al. Treatment of the clinical symptoms caused by osteoarthritis using nuclear magnetic resonance (MBST) in dogs a randomized trial-a pilot study. *Vet Med Austria* 2017;104:109. | 8, 9 |
| 11 | 11 | O | Oliva R, Jansen B, Benscheidt F, Sandbichler A.M, Egg M. Nuclear magnetic resonance affects the circadian clock and hypoxia-inducible factor isoforms in zebrafish. *Biol Rhythm Res* 2019;50(5):739-57. | 12 |
| 12 | 12 | O | Rad et al. Nuclear magnetic resonance treatment induces ß-NGF release from schwann cells and enhances the neurite growth of dorsal root ganglion neurons in vitro. *Cells* 2024;13(18):1544. | 3, 5, 10 (2x), 12 (2x) |
| 13 | 13 | O | Salfinger et al. Nuclear magnetic resonance therapy in lumbar disc herniation with lumbar radicular syndrome: effects of the intervention on pain intensity, health-related quality of life, disease-related disability, consumption of pain medication, duration of sick leave and MRI analysis. *Eur Spine J* 2015;24(6):1296-308. | 4, 5, 6, 11 (2x), 12 (2x), 13 (2x) |
| 14 | 14 | O | Steinecker-Frohnwieser et al. Nuclear magnetic resonance therapy modulates the miRNA profile in human primary OA chondrocytes and antagonizes inflammation in Tc28/2a cells. *Int J Mol Sci* 2021;22(11):5959. | 3, 5, 10 (2x), 12 (2x) |
| 15 | 15 | O | Steinecker-Frohnwieser et al. The therapeutic nuclear magnetic resonance changes the balance in intracellular calcium and reduces the interleukin-1β induced increase of NF-κB activity in chondrocytes. *Clin Exp Rheumatol* 2018;36(2):294-301. | 3, 6, 10 (2x), 12, 13 (2x) |
| 16 | 16 | O | Temiz-Artmann A, Linder P, Kayser P, Digel I, Artmann GM, Lücker P. NMR in vitro effects on proliferation, apoptosis, and viability of human chondrocytes and osteoblasts. *Methods Find Exp Clin Pharmacol* 2005;27(6):391-4. | 1, 2, 5, 8 (3x), 9 (3x), 12 (3x) |
| 17 | 17 | O | Thoeni et al. Therapeutic nuclear magnetic resonance and intermittent hypoxia trigger time dependent on/off effects in circadian clocks and confirm a central role of superoxide in cellular magnetic field effects. *Redox Biol* 2024;72:103152. | 4, 6, 11 (2x), 13 (2x) |
| 18 | 18 | O | Thöni et al. Quantum based effects of therapeutic nuclear magnetic resonance persistently reduce glycolysis. *iScience* 2022;25(12):105536. | 4, 6, 11, 13 |
| 19 | 19 | O | Thöni et al. Therapeutic nuclear magnetic resonance affects the core clock mechanism and associated hypoxia-inducible factor-1. *Chronobiol Int* 2021;38(8):1120-34. | 4, 6, 11 (2x), 13 (2x) |
| 20 | 1 | R | Krpan. A new concept of integrated holistic approch in treatment of chronic musculoskeletal diseases the "BAR" method. *Period Biol* 2015;117(1):119-24. | 12 |
| 21 | 2 | R | Krysiak-Zielonka. Regeneration of bone and cartilage tissue - new treatment and rehabilitation strategy. *Ortop Traumatol Rehabil* 2024;26(5):225-32. | 1, 2, 3, 5, 8 (2x), 9 (2x), 10 (2x), 12 (2x) |
| 22 | 3 | R | Schmidt et al. Magnetic resonance therapy in the treatment of osteoarthritis: A scoping review. *Radiography* 2021;27(3):968-75. | 1, 2, 7, 8 (2x), 9 (2x), 14 (2x) |
| 23 | 4 | R | Žnidarič et al. Potential of molecular biophysical stimulation therapy in chronic musculoskeletal disorders: a narrative review. *Eur J Transl Myol* 2023;33(4):11894. | 1, 2, 5, 7, 8 (2x), 9 (2x), 12 (2x), 14 (2x) |
| 24 | 1 | C | Kullich and Schwann. MBST (R) nuclear resonance therapy improves rehabilitation outcome in patients with low back pain. *Ann Rheum Dis* 2005;64;(Suppl. 3):519. | 8, 9 |
| 25 | 2 | C | Lohberger et al. Changes in the miRNA profile and hypoxic behaviour of human chondrocytes by therapeutic nuclear magnetic resonance therapy (NMRT). *Ann Rheum Dis* 2019;78(Suppl. 2):1514. | 10 (2x), 12 (2x) , 13 (2x) |
| 26 | 3 | C | Mohr et al. Beneficial effects of nuclear magnetic resonance therapy on liver regeneration. *Gastroenterol* 2021;59(1):e27. | 10, 12 |
| 27 | 4 | C | Peehal et al. Nuclear magnetic resonance therapy for knee joint osteoarthrosis: is there any clinical or radiological beneficial effect? A double blind randomised control study. *J Bone Joint Surg Br* 2011;93-B:308. | 12 |
| 28 | 5 | C | Steinecker-Frohnwieser al. Influence of NMR therapy on metabolism of osteosarcoma- and chondrosarcoma cell lines. *Bone* 2009;44(Suppl. 2):S295. | 8, 9, 10, 12 (2x) |
| 29 | 1 | N | A trial of mindfulness-based skills training groups versus a waiting list control period for parents of obsessive-compulsive disorder (OCD)-affected youth. *Clinicaltrials.gov* 2017;no pagination. | 8, 9 |
| 30 | 2 | N | Abbo et al. Mechanism-based-susceptibility testing (MBST) using disc diffusion assays (DDA) to guide treatment of multidrug- and extensively drug-resistant pseudomonas aeruginosa (MDR-XDR-PA) in a cystic fibrosis (CF) lung transplant recipient; Are we ready for combination therapy vs. MDR-XDR-PA? *Open Forum Infect Dis* 2018;5(Suppl. 1):S714. | 8 (3x), 9 (3x) |
| 31 | 3 | N | Abdi et al., The effect of repeated flexion-based exercises versus extension-based exercises on the clinical outcomes of patients with lumbar disk herniation surgery: a randomized clinical trial. *Neurol Res* 2023;45(1):28-40. | 1, 2, 8 (2x) |
| 32 | 4 | N | Abramova and Belichenko. The clinical aspect of the joint use of magnetic resonance tomography of the brain and magnetic resonance angiography of the extra- and intracranial arteries in patients with arterial hypertension. [in Russian] *Ter Arkh* 1996;68(9):26-31. | 3, 10 (3x) |
| 33 | 5 | N | Akbari et al. Systems thinking, causal loop diagram, and systems dynamic in public health challenges: navigating long COVID syndrome and sense of smell in LGBTQIA+ communities. *Public Health Chall* 2024;3(3):e70004. | 1, 8 (3x) |
| 34 | 6 | N | Aksoy et al. Proposal for an expanded "R" classification: impact of positive surgical margin length on biochemical recurrence after robotic radical prostatectomy. *J Clin Med* 2025;14(12):4310. | 4 |
| 35 | 7 | N | Aqeele et al. Determination of the effective dose of curcumin alone and in combination with antimicrobial peptide CM11 on promastigote forms of Iranian strain of L. major (MRHO / IR / 75 / ER). *Arch Razi Inst* 2019;74(4):413-22. | 1, 2 |
| 36 | 8 | N | Aqeele et al. Evaluation of curcumin and CM11 peptide alone and in combination against amastigote form of Iranian strain of L. major (MRHO/IR75/ER) in vitro. *Exp Parasitol* 2021;229:108151. | 1, 2 |
| 37 | 9 | N | Arasaradnam et al. TNM&R staging in colorectal cancer: has the two week wait for patients made any difference? *Gut* 2002;50;(Suppl. 2):A48. | 11 (2x) |
| 38 | 10 | N | Aue W.P. Topical nuclear magnetic resonance--a non-invasive probe for biochemical measurements in living organisms. [in German] *Radiologe* 1983;23(8):357-60. | 4, 11 (2x) |
| 39 | 11 | N | Aupaix et al. Evaluation of a new protocol for rapid identification of Streptococcus pneumoniae in blood cultures using the modified bile solubility test: Gram staining is still standing. *J Clin Microbiol* 2025;63(1):e0122224. | 1, 8 (2x) |
| 40 | 12 | N | Austerberry et al. The effect of charge mutations on the stability and aggregation of a human single chain Fv fragment. *Eur J Pharm Biopharm* 2017;115:18-30. | 11 |
| 41 | 13 | N | Badoux et al. Change of carrier density at the pseudogap critical point of a cuprate superconductor. *Nature* 2016;531(7593):210-4. | 11 |
| 42 | 14 | N | Bahcivan et al. A single-session mindfulness-based swinging technique vs. cognitive disputation intervention among women with breast cancer: A pilot randomised controlled study examining the efficacy at 8-week follow-up. *Front Psychol* 2022;13:1007065. | 1, 2, 8, 9 |
| 43 | 15 | N | Bahcivan et al. Efficacy of new mindfulness-based swinging technique intervention: a pilot randomised controlled trial among women with breast cancer. *Front Psychol* 2022;13:863857. | 1, 2, 8, 9 |
| 44 | 16 | N | Baumgarten et al. Analysis of surgeon biometrics during open and robotic radical cystectomy with electromyography and motion capture analysis. *Int Braz J Urol* 2020;46(1):138. | 1, 2, 8 |
| 45 | 17 | N | Begum et al. Active Rho kinase (ROK-alpha) associates with insulin receptor substrate-1 and inhibits insulin signaling in vascular smooth muscle cells. *J Biol Chem* 2002;277(8):6214-22. | 8 |
| 46 | 18 | N | Belopitova et al. Glioma of the optical nerve diagnostic and differential diagnostic problems. *Pediatriya* 1997;36(3):50-2+5. | 10 |
| 47 | 19 | N | Belopitova et al. Nuclear magnetic resonance tomography in resistant epileptic syndromes in childhood - New possibilities for the disclosure of cerebral pathology. *Pediatriya* 1998;38(3):27-30. | 10 |
| 48 | 20 | N | Beng et al. Mindfulness-based supportive therapy (MBST): proposing a palliative psychotherapy from a conceptual perspective to address suffering in palliative care. *Am J Hosp Palliat Care* 2015;32(2):144-60. | 1, 2, 8 (3x), 9 (3x) |
| 49 | 21 | N | Bialik et al. Multifocal aseptic bone necrosis after COVID-19: a clinical case. *Aging Clin Exp Res* 2022;34(Suppl. 1):S353-4. | 12 (3x) |
| 50 | 22 | N | Bilderback et al. Phosphoinositide 3-kinase regulates crosstalk between Trk A tyrosine kinase and p75NTR-dependent sphingolipid signaling pathways. *J Neurochem* 2001;76(5):1540-51. | 8 |
| 51 | 23 | N | Burton et al. Identification of an ordered compact structure within the recombinant bovine fibrinogen alpha C-domain fragment by NMRT. *Biochemistry* 2006;45;(7):2257-66. | 10 |
| 52 | 24 | N | Cainelli et al. Chemo- and enzyme-catalyzed reactions revealing a common temperature-dependent dynamic solvent effect on enantioselectivity. *Helv Chim Acta* 2003;86(11):3548-59. | 11 |
| 53 | 25 | N | Cainelli et al. Chiral aldehydes in hydrocarbons: diastereoselective nucleophilic addition, NMR, and CD spectroscopy reveal dynamic solvation effects. *Chirality* 2004;16(1):50-6. | 4, 11 |
| 54 | 26 | N | Cainelli et al. Dynamic solvation effects on the endo/exo selectivity of the Diels-Alder reaction. *Tetrahedron Lett* 2003;44(1):93-6. | 11 (2x) |
| 55 | 27 | N | Cao et al. MBST-Driven 4D-CBCT reconstruction: leveraging swin transformer and masking for robust performance. *Comput Methods Programs Biomed* 2025:262:108637. | 1, 8 (2x) |
| 56 | 28 | N | Cheetham et al. How do nuclear medicine radiographers and technologists understand the concept of patient care? *Nucl Med Commun* 2021;42(10):1172-3. | 10 |
| 57 | 29 | N | Chen et al. Artificial cavernosa-like tissue based on multibubble matrigel and a human corpus cavernous fibroblast scaffold. *Asian J Androl* 2024;26(3):260-7. | 8, 9 |
| 58 | 30 | N | Chen et al. Tunable chiral magneto-transport through band structure engineering in magnetic topological insulators Mn(Bi1-x Sb x )2Te4. *Sci Adv* 2025;11(20):eadt6084. | 8 (2x) |
| 59 | 31 | N | Cheung et al. Carcinoma of the breast: measurement and the management of treatment. I. The value of the data. *Br J Radiol* 1991;64(757):29-36. | 1, 2, 8 (4x), 9 (4x) |
| 60 | 32 | N | Cornil et al. Organizing effects of sex steroids on brain aromatase activity in quail. PLoS ONE 2011;6(4):e19196. | 1, 8 (2x) |
| 61 | 33 | N | da Silva Temperini et al. Insecticidal activity in vitro of the essential oil of Pogostemon cablin against Ctenocephalides felis felis. *Braz J Vet Med* 2022;44:e003422. | 1 |
| 62 | 34 | N | Dickens et al. Acute stress differentially affects aromatase activity in specific brain nuclei of adult male and female quail. *Endocrinology* 2011;152(11):4242-51. | 8 |
| 63 | 35 | N | Dickens et al. Brain aromatase and circulating corticosterone are rapidly regulated by combined acute stress and sexual interaction in a sex-specific manner. *J Neuroendocrinol* 2012;24(10):1322-34. | 8 |
| 64 | 36 | N | Dickens et al. Neurochemical control of rapid stress-induced changes in brain aromatase activity. *J Neuroendocrinol* 2013;25(4):329-39. | 8 |
| 65 | 37 | N | Dogan Kuday et al. Relationship between emotional intelligence and disaster response self-efficacy: A comparative study in nurses. *Int Emerg Nurs* 2023;70:101319. | 3, 10 (2x) |
| 66 | 38 | N | Efficacy of mindfulness based swinging technique (MBST) for improving anxiety, stress, hopelessness and self efficacy among breast cancer patients in treatment. *Clinicaltrials.gov* 2019;no pagination. | 8, 9 |
| 67 | 39 | N | El-Dawlatly et al. Anesthetic implications for video assisted thoracoscopic thymectomy in myasthenia gravis. *Middle East J Anesthiol* 2005;18(2):339-45. | 3, 10 (3x) |
| 68 | 40 | N | El-Fayoumy et al. Evaluation of antioxidant and anticancer activity of crude extract and different fractions of Chlorella vulgaris axenic culture grown under various concentrations of copper ions. *BMC Complement Med Ther* 2021;21(1):51. | 3, 10 (2x) |
| 69 | 41 | N | El-Tahan and Regal. Target-controlled infusion of remifentanil without muscle relaxants allows acceptable surgical conditions during thoracotomy performed under sevoflurane anesthesia. *J Cardiothorac Vasc Anesth* 2015;29(6):1557-66. | 3, 10 (2x) |
| 70 | 42 | N | Espin et al. Creating a progress-monitoring system in reading for middle-school students: tracking progress toward meeting high-stakes standards. *LDRP* 2010;25(2):60-75. | 8 |
| 71 | 43 | N | Evtushenko and Derevianko. The role of nuclear magnetic resonance monitoring in early clinical-instrumental diagnosis of multiple sclerosis. [in Russian] *Zh Nevrol Psikhiatr Im S S Korsakova* 1998;98(12):29-31. | 3, 10 (3x) |
| 72 | 44 | N | Farag et al. "TNMR" versus "TNM" in the staging of colorectal cancer. *Colorectal Dis* 2014;16(Suppl. 3):67. | 11 (2x) |
| 73 | 45 | N | Faridi et al. Experimental assessment of microwave ablation computational modeling with MR thermometry. *Med Phys* 2020;47(9):3777-88. | 10 |
| 74 | 46 | N | Festinger. The significanee of difference between means without reference to the frequency distribution function. *Psychometrika* 1946;11(2):97-106. | 8 (2x) |
| 75 | 47 | N | Fu et al. BST-2/Tetherin is involved in BAFF-enhanced proliferation and survival via canonical NF-[kappa]B signaling in neoplastic B-lymphoid cells. *Exp Cell Res* 2021;398(1):112399. | 8 |
| 76 | 48 | N | Gerek et al. The effectiveness of fiberoptic endoscopic swallow study and modified barium swallow study techniques in diagnosis of dysphagia [in Turkish]. *Kulak Burun Bogaz Ihtis Derg* 2005;15(5-6):103-11. | 1, 8 (2x) |
| 77 | 49 | N | Giratallah et al. Nicotine metabolite ratio: comparison of the three urinary versions to the plasma version and nicotine clearance in three clinical studies. *Drug Alcohol Depend* 2021;223:108708. | 10 (2x) |
| 78 | 50 | N | Goychuk. Viscoelastic subdiffusion: from anomalous to normal. *Phys Rev E Stat Nonlin Soft Matter Phys* 2009;80(4 Pt 2):046125. | 3, 10 (2x) |
| 79 | 51 | N | Greco et al. Mind-body skills training and supportive counseling for depression in SLE: positive effects in a randomized controlled trial. *Arthritis Rheum* 2016;68(Suppl. 10):1386-1387. | 8, 9 |
| 80 | 52 | N | Healthy relationships training study: comparing the efficacy of a mindfulness-based skills training to a psychoeducational intervention at reducing dating violence on college campuses. *Clinicaltrials.gov* 2019;no pagination. | 8, 9 |
| 81 | 53 | N | Hu et al. Effect of freeze-thaw cycles on mechanical performance of loess soil stabilized with nano magnesium oxide. *PLoS ONE* 2025;20(4):e0319909. | 3, 10 (2x) |
| 82 | 54 | N | Hwang and Lee. Comparison of efficacy of reverse moxifloxacin-based sequential therapy and moxifloxacin-based sequential therapy as first-line eradication regimen for helicobacter pylori infection. *Digestion* 2017;95(1):100-101. | 8 (3x), 9 (3x) |
| 83 | 55 | N | Hwang et al. Comparison of efficacy of bismuth containing quintet therapy and moxifloxacinbased sequential therapy as first- line eradication regimen for Helicobacter Pylori infection. *Helicobacter* 2016;21(Suppl. 1):144. | 8, 9 |
| 84 | 56 | N | Hwang et al. Efficacy of moxifloxacin-based sequential and hybrid therapy for first-line Helicobacter pylori eradication. *World J Gastroenterol* 2015;21(35):10234-10241. | 1, 2, 8 (2x), 9 (2x) |
| 85 | 57 | N | Jeong et al. Neuromuscular retraining therapy combined with preceding botulinum toxin A injection for chronic facial paralysis. *Acta Oto-Laryngol* 2023;143(5):446-51. | 3, 10 (2x) |
| 86 | 58 | N | Jhou et al. The mesopontine rostromedial tegmental nucleus: A structure targeted by the lateral habenula that projects to the ventral tegmental area of Tsai and substantia nigra compacta. *J Comp Neurol* 2009;513(6):566-96. | 8 |
| 87 | 59 | N | Jia et al. Ethylene-induced hydrogen sulfide negatively regulates ethylene biosynthesis by persulfidation of ACO in tomato under osmotic stress. *Front Plant Sci* 2018;9:1517. | 1, 8 |
| 88 | 60 | N | Johnson et al. The management of individual breast cancers. *Breast* 1995;4(2):100-11. | 8 (2x), 9 (2x) |
| 89 | 61 | N | Joshi et al. Effect of a single-session mindfulness-based swinging technique (MBST) intervention on emotional distress in cancer patients undergoing PET-CT scan: a randomized controlled trial. *J Integr Complement Med* 2024;30(10):961-9. | 1, 2, 8 (2x) |
| 90 | 62 | N | Kazemi et al. Comparing the effectiveness of mindfulness based schema therapy and transdiagnostic intervention on cognitive fusion with illness and posttraumatic avoidance in women with breast cancer: a semi-experimental study. *J Mazandaran Univ Med Sci* 2023;33(220):54-65. | 8, 9 |
| 91 | 63 | N | Kebert et al. Ectomycorrhizal fungi modulate pedunculate oak's heat stress responses through the alternation of polyamines, phenolics, and osmotica content. *Plants* 2022;11(23):3360. | 3 |
| 92 | 64 | N | Khalid et al. Performance evaluation of membrane-based septic tank and its reuse potential for irrigating crops. *Water* *Environ Res* 2017;89(8):744-51. | 1, 8 (2x) |
| 93 | 65 | N | Kim and Speece. Reactor configuration-part II comparative process stability and efficiency of thermophilic anaerobic digestion. *Environ Technol* 2002;23(6):643-54. | 4, 11 (3x) |
| 94 | 66 | N | Kim et al. Comparison of efficacy of bismuth-containing quintet therapy and moxifloxacin-based sequential therapy as first-line eradication regimen for Helicobacter pylori infection. *United Eur Gastroenterol J* 2019;7(Suppl. 8):731. | 8, 9 |
| 95 | 67 | N | Kim et al. Comparing efficacy of Helicobacter pylori eradication regimens: bismuth containing quintet therapy vs moxifloxacin based sequential therapy as first-line eradication regimen. *Helicobacter* 2020;25(Suppl. 1): no pagination. | 8, 9 |
| 96 | 68 | N | Lange and Seyyedi. Evidence of a Lyme borreliosis infection from the viewpoint of laboratory medicine. *Int J Med* *Microbiol* 2002;291(Suppl 33):120-4. | 1, 8 (2x) |
| 97 | 69 | N | Lee et al. Comparison of efficacy of bismuth containing quintet therapy and moxifloxacin-based sequential therapy as first-line eradication regimen for helicobacter pylori infection. *Digestion* 2018;97(1):120. | 8, 9 |
| 98 | 70 | N | Lee et al. Comparison of efficacy of bismuth containing quintet therapy and moxifloxacin-based sequential therapy as first-line eradication regimen for helicobacter pylori infection. *Dig Endosc* 2020;32(Suppl. 1):197. | 8, 9 |
| 99 | 71 | N | Lennon-Maslin and Quaiser-Pohl. "It's different for girls!" The role of anxiety, physiological arousal, and subject preferences in primary school children's math and mental rotation performance. *Behav Sci* 2024;14(9):809. | 3, 10 |
| 100 | 72 | N | Li et al. Seed transmission of three viruses in two pear rootstock species Pyrus betulifolia and P. calleryana. *Viruses* 2022;14(3):599. | 3, 10 (2x) |
| 101 | 73 | N | Löhrke et al. Spontaneous tumors and lifespan of female NMRI mice of the outbred stock Sut:NMRT during a lifetime study. *J Cancer Res Clin Oncol* 1984;108(2):192-6. | 3, 10 (2x) |
| 102 | 74 | N | Loughmiller et al. An evaluation of differences in mean body surface temperature with infrared thermography in growing pigs fed different dietary energy intake and concentration. *J Appl Anim Res* 2005;28;(2):73-80. | 8 |
| 103 | 75 | N | Loughmiller et al. Relationship between mean body surface temperature measured by use of infrared thermography and ambient temperature in clinically normal pigs and pigs inoculated with Actinobacillus pleuropneumoniae. *Am J Vet Res* 2001;62(5):676-81. | 1, 8 (3x) |
| 104 | 76 | N | Lusk Monagle et al. New graduate nurse experiences in clinical judgment: what academic and practice educators need to know. *Nurs Educ Perspect* 2018;39(4):201-7. | 1 |
| 105 | 77 | N | Malham et al. Porous cage macro-topography improves early fusion rates in anterior cervical discectomy and fusion. *Surg Res Pract* 2024;2024:8452050. | 3, 10 (2x) |
| 106 | 78 | N | Matsukawa et al. Changes in the density of activated neurons in the medial part of the bed nucleus of stria terminalis following electrical stimulation in the mice olfactory bulb. *Neurosci Res* 2010;68(Suppl. 1):e389. | 8 |
| 107 | 79 | N | Matsukawa et al. Rose odor can innately counteract predator odor. *Brain Res* 2011;1381:117-23. | 1, 8 (3x) |
| 108 | 80 | N | Matsumoto et al. In vivo pharmacokinetic analysis utilizing non-targeted and targeted mass spectrometry and in vitro assay against transient receptor potential channels of maobushisaishinto and its constituent asiasari radix. *Molecules* 2020;25(18):4283. | 1, 2, 8 (2x) |
| 109 | 81 | N | McGinty et al. Emerging, reemerging, and forgotten brain areas of the reward circuit: Notes from the 2010 Motivational Neural Networks conference. *Behav Brain Res* 2011;225(1):348-57. | 8 |
| 110 | 82 | N | McLaughlin et al. Comparison of performance, clinical chemistry, and carcass characteristics of finishing lambs treated with recombinant ovine or bovine somatotropins. *J Anim Sci* 1993;71(6):1453-63. | 1 |
| 111 | 83 | N | Mendes and Silveira PF. The interrelationship between leukotriene B4 and leukotriene-A4-hydrolase in collagen/adjuvant-induced arthritis in rats. *Biomed Res Int* 2014;2014:730421. | 1 |
| 112 | 84 | N | Menezes et al. Deep Q-Managed: a new framework for multi-objective deep reinforcement learning. *Front Artif Intell* 2025;8:1683323. | 1, 8 |
| 113 | 85 | N | Miller et al. Hybrid positron emission tomography/magnetic resonance imaging in arrhythmic mitral valve prolapse. *JAMA Cardiol* 2020;5(9):1000-5. | 11 |
| 114 | 86 | N | Mind body therapy for the treatment of chronic pain. *Clinicaltrials.gov* 2019;no pagination. | 8, 9 |
| 115 | 87 | N | Minelli et al. Neuro-motor reaction time in young non-athletes in age of development. [in Italian] *Med Sport* 1998;51(1):1-11. | 10 (2x) |
| 116 | 88 | N | Miranda et al. Contactless body temperature assessment for signalling humane endpoints in a mouse model of sepsis. *Anim Welf* 2025;34:e13. | 1, 8 (2x) |
| 117 | 89 | N | Monini et al. Combined protocol for treatment of secondary effects from facial nerve palsy. *Acta Otolaryngol* 2011;131(8):882-6. | 3, 10 (2x) |
| 118 | 90 | N | Mura et al. Organizational effects of DDE on brain vasotocin system in male Japanese quail. *Neurotoxicol* 2009;30(3):479-84. | 8 |
| 119 | 91 | N | Murakami et al. Stress-related activities induced by predator odor may become indistinguishable by hinokitiol odor. *Neuroreport* 2012;23(18):1071-1076. | 8 (2x) |
| 120 | 92 | N | Ni et al. 4D-CBCT reconstruction with mask-based swin transformer network for improving image quality. *Med Phys* 2024;51(9):6722. | 8 |
| 121 | 93 | N | Nuclear magnetic resonance therapy in knee osteoarthrosis: a double blind ranomised controlled trial. *Clinicaltrials.gov* 2012;no pagination. | 8, 9, 10, 12 |
| 122 | 94 | N | Nuesslein-Hildesheim and Schmidt I. Is the circadian core temperature rhythm of juvenile rats due to a periodic blockade of thermoregulatory thermogenesis? *Pflugers Arch* 1994;427(5-6):450-4. | 4, 11 (3x) |
| 123 | 95 | N | Oku. Network analysis of kampo formulas based on crude drug composition and indications. *Tradit Kampo Med* 2019;6(3):139-47. | 8 |
| 124 | 96 | N | Olmos et al. Comparison of two coprological methods for the diagnosis of Eurytrema ssp. in cattle and sheep. *J Helminthol* 2022;96:e53. | 1, 8 (2x) |
| 125 | 97 | N | Omonkhua and Onoagbe. Effect of irvingia gabonensis total saponin fractions on blood glucose, lipid profile and oxidative status of normal male rats. *Planta Med* 2014;80(16):P2B10. | 11 |
| 126 | 98 | N | Otake et al. Colloidal particle in suspensions of maobushisaishinto extract granules enhances drug intestinal penetration. *Chem Pharm Bull* 2024;72(12):1055-60. | 1, 8 (2x) |
| 127 | 99 | N | Patel et al. Analysis of surgeon biometrics during open and robotic radical cystectomy. *J Endourol* 2017;31(Suppl. 2):A248-9. | 8 |
| 128 | 100 | N | Peters et al. Changes in mean body surface temperature (MBST) of the Neonate After Feeding^+^ 1097. *Pediatr Res* 1998;43(4):188. | 8 (3x) |
| 129 | 101 | N | Petrovs'kyĭ et al. Kliniko-renthenolohichna otsinka zmin stopy u khvorykh na tsukrovyĭ tiabet [Clinico-radiological estimation of the foot changes in patients with diabetes mellitus]. *Klin Khir* 2001;(8):37-9. | 3, 10 (2x) |
| 130 | 102 | N | Phelps et al. Individual differences in vasopressin V1A receptor expression, promoter structure and social behavior. *Soc Neurosci Abstract* 2002;2002:89.1. | 8 |
| 131 | 103 | N | Pijoan. Secretion of hydrogen peroxide by some common pig mycoplasmas. *Vet Rec* 1974;95(10):216-7. | 8 |
| 132 | 104 | N | Pourmomeny et al. Neuromuscular retraining versus BTX-A injection in subjects with chronic facial nerve palsy, a clinical trial. *Iran J Otorhinolaryngol* 2021;33(116):151-5. | 3, 10 (2x) |
| 133 | 105 | N | Purohit et al. Case series: transient neonatal macular retinoschisis - a developmental phenomenon in term infants. *Eye* 2025;39(18):3359-64. | 4, 11 (2x) |
| 134 | 106 | N | Raji et al. IL-7R α polymorphisms in 60 Iranian multiple sclerosis patients. *Iran J Neurol* 2012;11(1):6-11. | 1, 8 |
| 135 | 107 | N | Read et al. Relationships between field-based measures of strength and power and golf club head speed. *J Strength Cond Res* 2013;27(10):2708-13. | 1, 8 (4x) |
| 136 | 108 | N | Rebuffat et al. Tricholongins BI and BII 19-residue peptaibols from Trichoderma-longibrachiatum solution structure from two-dimensional NMR spectroscopy. *Eur J Biochem* 1991;201;(3):661-74. | 10 |
| 137 | 109 | N | Reducing depressive symptoms in systemic lupus erythematosus. *Clinicaltrials.gov* 2010;no pagination. | 8, 9 |
| 138 | 110 | N | Rieger-Hug and Stirm. Comparative study of host capsule depolymerases associated with Klebsiella bacterio phages. *Virology* 1981;113;(1):363-78. | 8 |
| 139 | 111 | N | Romcevich et al. Mind-body skills training for resident wellness: a pilot study of a brief mindfulness intervention. *J Med Educ Curric Dev* 2018;5:2382120518773061. | 1, 8 |
| 140 | 112 | N | Safaei et al. A simple and accurate PCR method for detection of genetically modified rice. *J Environ Health Sci Eng* 2019;17(2):847-51. | 1, 8 |
| 141 | 113 | N | Saint-Jalmes et al. Virtual phantom magnetic resonance imaging (ViP MRI) on a clinical MRI platform. *Med Phys* 2018;45(1):250-7. | 11 |
| 142 | 114 | N | Samborski et al. Adaptation of fracture mechanics methods for quality assessment of tungsten carbide cutting inserts. *Materials* 2021;14(13):3441. | 4, 11 |
| 143 | 115 | N | Sasidharanpillai. Triborate formation constants and polyborate speciation under hydrothermal conditions by Raman spectroscopy using a titanium/sapphire flow cell. *J Phys Chem B* 2019;123(24):5147-59. | 1, 8 (2x) |
| 144 | 116 | N | Sato et al. [Effects of half-sized secretory leukocyte protease inhibitor and Chinese traditional medicines, yokuinin and mao-bushi-saishin-to, on therapeutic efficacies of benzoxazinorifamycin KRM-1648 against Mycobacterium avium complex infection induced in mice]. *Kekkaku* 1998;73(8):501-6. Japanese. | 1, 2, 8 (2x), 9 (2x) |
| 145 | 117 | N | Sayag and Yahr. Unilateral lesions of the sexually dimorphic area SDA of the gerbil hypothalamus disrupt male sexual behavior when combined with contralateral lesions of the medial amygdala-amgydalohippocampal area MA-AHI or medial bed nucleus of the stria terminalis MBST. *Soc Neurosci Abstracts* 1991;17;(1-2):1061. | 8 |
| 146 | 118 | N | Shen et al. Strong deaggregating effect of a novel polyamino resorcinarene surfactant on gold nanoaggregates under microwave irradiation. *Langmuir* 2008;24(22):13161-7. | 4, 11 |
| 147 | 119 | N | Shibata et al. [Effect of mao-bushi-saishin-to (MBST), a formula of Chinese medicines, on 48 hr homologous passive cutaneous anaphylaxis in rats]. *Arerugi* 1995;44(9):1167-75. Japanese. | 1, 8 |
| 148 | 120 | N | Shibata et al. [Effects of mao-bushi-saishin-to (MBST) on experimental allergic models in rats]. *Arerugi* 1995;44(10):1234-40. Japanese. | 1, 2, 8 (3x) |
| 149 | 121 | N | Shibata et al. Effect of mao-bushi-saishin-to (MBST), a formula of Chinese medicines, on 48 hr homologous passive cutaneous anaphylaxis in rats. *Jap J Allergol* 1995;44;(9):1167-75. | 8 (2x) |
| 150 | 122 | N | Shibata et al. l-ephedrine is a major constituent of mao-bushi-saishin-to, one of the formulas of Chinese medicine, which shows immediate inhibition after oral administration of passive cutaneous anaphylaxis in rats. *Inflamm Res* 2000;49(8):398-403. | 1, 8 (3x) |
| 151 | 123 | N | Shimizu et al. [Effects of the Chinese traditional medicines "mao-bushi-saishin-to" and "yokuinin" on the antimycobacterial activity of murine macrophages against Mycobacterium avium complex infection]. *Kekkaku* 1999;74(9):661-6. Japanese. | 1, 2, 8 (2x) |
| 152 | 124 | N | Shimizu et al. [Therapeutic effects of benzoxazinorifamycin KRM-1648 administered alone or in combination with glycyrrhizin against Mycobacterium avium complex infection in mice]. *Kekkaku* 1999;74(8):617-21. Japanese. | 1, 2, 8 (2x) |
| 153 | 125 | N | Shimizu et al. Effects of the Chinese traditional medicine mao-bushi-saishin-to on therapeutic efficacy of a new benzoxazinorifamycin, KRM-1648, against Mycobacterium avium infection in mice. *Antimicrob Agents Chemother* 1999;43(3):514-9. | 1, 2, 8 (3x) |
| 154 | 126 | N | Shirmohammad et al. Evaluation of nanonanoliposomal curcumin on cutaneous leishmaniasis skin lesions caused by Leishmania major in BALB/c mice. *Iran J Parasitol* 2024;19(2):238-46. | 1 |
| 155 | 127 | N | Simkin et al. Meditation/mindfulness: use in clinical practice. *J Am Acad Child Adolesc Psychiatry* 2017;56(10):S128. | 8, 9 |
| 156 | 128 | N | Slavov et al. Anesthetic considerations for H-reflex monitoring during surgical treatment of spastic disorders. *Anesthesiology Abstracts of Scientific Papers Annual Meeting* 2002;(2000):Abstract No. 287. | 11 |
| 157 | 129 | N | Spees et al. Rat brain global ischemia-induced diffusion changes revisited: biophysical modeling of the water and NAA MR "diffusion signal". *Magn Reson Med* 2022;88(3):1333-46. | 11 |
| 158 | 130 | N | Swierczek-Jereczek et al. Time-scale synchronisation of oscillatory responses can lead to non-monotonous R-tipping. *Sci Rep* 2023;13(1):2104. | 3, 10 (2x) |
| 159 | 131 | N | Taghavi et al. Ultrasound super-resolution imaging with a hierarchical Kalman tracker. *Ultrasonics* 2022;122:106695. | 1 |
| 160 | 132 | N | Taki et al. Prediction of final tumor response to preoperative chemotherapy by Tc-99m MIBI imaging at the middle of chemotherapy in malignant bone and soft tissue tumors: comparison with Tl-201 imaging. *J Orthop Res* 2008;26(3):411-8. | 1, 2, 8 (3x) |
| 161 | 133 | N | Tan et al. Mindfulness: a new paradigm of psychosocial care in the palliative care setting in southeast asia. *Ann Acad Med Singap* 2017;46(9):339-46. | 1, 2, 8 (2x), 9 (2x) |
| 162 | 134 | N | Tan et al. Mindfulness-based supportive therapy on reducing suffering in patients with advanced cancer: randomised controlled trial. *BMJ Support Palliat Care* 2024;13(e3):e1117-25. | 1, 2, 8 (3x), 9 (3x) |
| 163 | 135 | N | Tanemura et al. A case of successful fetal therapy for congenital chylothorax by intrapleural injection of OK-432. *Ultrasound Obstet Gynecol* 2001;18(4):371-5. | 4 |
| 164 | 136 | N | Target-controlled infusion of remifentanil without muscle relaxant allows acceptable surgical conditions during thoracotomy. *Clinicaltrials.gov* 2014;no pagination. | 10 |
| 165 | 137 | N | The effect of fatigue caused by search and rescue efforts in the debris on the quality of cardiopulmonary resuscitation: a randomized controlled trial. *Clinicaltrials.gov* 2024;no pagination. | 10 |
| 166 | 138 | N | The immediate effect of mindfulness-based supportive therapy (MBST) vs supportive listening on palliating suffering in palliative care cancer patients: a randomised controlled trial. *Clinicaltrials.gov* 2021;no pagination. | 8, 9 |
| 167 | 139 | N | The mindfulness, incontinence and sexual function treatment study. *Clinicaltrials.gov* 2016;no pagination. | 8, 9 |
| 168 | 140 | N | Tian et al. Synthesis of [3α-(3) H] 17α-hydroxy pregnenolone and [3α-(3) H] pregnenolone. *J Labelled Comp Radiopharm* 2014;57(1):1-11. | 4 |
| 169 | 141 | N | Trageser et al. Outcomes of dogs with thymoma treated with intensity modulated stereotactic body radiation therapy or non-modulated hypofractionated radiation therapy. *Vet Comp Oncol* 2022;20(2):491-501. | 3, 10 (4x) |
| 170 | 142 | N | Tsunomura and Tokumoto. Man-made electromagnetic noises causing difficulty in geomagnetic and geoelectric observations in city area. *Biomed Pharmacother* 2005;59(Suppl 1):S15-9. | 4 |
| 171 | 143 | N | Vahdani et al. Quantitative and qualitative analysis of three DNA extraction methods from soybean, maize, and canola oils and investigation of the presence of genetically modified organisms (GMOs). *Food Chem* 2024;8:100201. | 1, 8 (2x) |
| 172 | 144 | N | Van Den Bergh et al. Muscle glycogen recovery after exercise during glucose and fructose intake monitored by 13C-NMR. *J Appl Physiol* 1996;81(4):1495-500. | 4, 11 |
| 173 | 145 | N | Vassilev et al. The occlusion of secondary small branches arising in region of coronary bifurcation lesions increases restenosis rates after bifurcation stenting. The link between periprocedural ischemia and instent. *Eur Heart J* 2013;34(Suppl. 1):216. | 8 |
| 174 | 146 | N | Voldstad et al. The effect of mindfulness interventions on couple relationship satisfaction: a systematic review and meta-analysis. *J Consult Clin Psychol* 2025;93(6):427-42. | 8, 9 |
| 175 | 147 | N | Voss et al. Mindfulness training in students leads to a reduction in physiological stress levels. *Biomed Tech* 2019;64(2):S75. | 8 |
| 176 | 148 | N | Voss et al. Mindfulness-based student training improves vascular variability associated with sustained reductions in physiological stress response. *Front Public Health* 2022;10:863671. | 1, 2, 8 (2x) |
| 177 | 149 | N | Voss et al. Mindfulness-based student training leads to a reduction in physiological evaluated stress. *Front Psychol* 2020;11:645. | 1, 8 |
| 178 | 150 | N | Wakabayashi et al. Prognostic value of 99mTc-MIBI performed during middle course of the preoperative chemotherapy in patients with malignant bone and soft tissue tumors. *Eur J Nucl Med Mol Imaging* 2010;37(Suppl. 2):S252-S253. | 8 |
| 179 | 151 | N | Wakabayashi et al. Prognostic value of Tc-99m-MIBI performed during middle course of preoperative chemotherapy in patients with malignant bone and soft-tissue tumors. *Clin Nucl Med* 2012;37(1):1-8. | 1, 2, 8 (4x) |
| 180 | 152 | N | Wang and Li. Effect of internal interface layer on dielectric properties of doped Ba0.6Sr0.4TiO3thin films and its simulation in filters. J Phys Condens Matter 2023;35(36):no pagination. | 1, 8 (2x) |
| 181 | 153 | N | Wang and Li. Effect of zinc and magnesium ion doping on leakage current behavior of Ba0.6Sr0.4TiO3 thin film. *RSC* *Adv* 2024;14(43):31746-31755. | 1, 8 |
| 182 | 154 | N | Xie et al. Achieving high energy density and low loss in PVDF/BST nanodielectrics with enhanced structural homogeneity. *ACS Appl Mater Interfaces* 2018;10(34):29038-47. | 1, 8 (2x) |
| 183 | 155 | N | Yao et al. A sensitive nested multiplex RT-PCR assay for the simultaneous detection of three common viruses infecting pear plants. *J Virol Methods* 2019;263:105-110. | 3, 10 (2x) |
| 184 | 156 | N | Yeh et al. Lectin-triggered aggregation of glyco-gold nanoprobes for activity-based sensing of hydrogen peroxide by the naked eye. *Chem Asian J* 2021;16(21):3462-8. | 1, 8 (2x) |
| 185 | 157 | N | Yotova et al. Diagnostics and treatment of resistant forms of epilepsy in childhood. *Pediatriya* 1997;36(3):39-43+4. | 10 |
| 186 | 158 | N | Yousefi et al. Molecular detection of Anaplasma phagocytophilum as a zoonotic agent in owned and stray dogs in Tehran, Iran. *Arch Razi Inst* 2019;74(1):33-8. | 1, 8 (3x) |
| 187 | 159 | N | Zheng et al. Concomitant chemoradiation followed by radical surgery for locally advanced cervical cancer patients: Preliminary results from a randomized, controlled trial. *Chin J Evid Based Med* 2017;17(1):1-6. | 10 |
